# Realization of a Photoelectrochemical Cascade for the Generation of Methanol: A Liquid Solar Fuel

**DOI:** 10.1021/acs.energyfuels.4c04779

**Published:** 2024-12-23

**Authors:** Thomas Chan, Calton J. Kong, Grace A. Rome, Darci K. Collins, Alex J. King, Rajiv Ramanujam Prabhakar, Sarah A. Collins, Michelle S. Young, Mickey J. Wilson, Myles A. Steiner, Adele C. Tamboli, Emily L. Warren, Clifford P. Kubiak, Joel W. Ager, Ann L. Greenaway

**Affiliations:** 1 Chemical Sciences Division, Lawrence Berkeley National Laboratory, Berkeley, California 94720, United States; 2 Materials Sciences Division, Lawrence Berkeley National Laboratory, Berkeley, California 94720, United States; 3 Department of Materials Science and Engineering, University of California, Berkeley, California 94720, United States; 4 Department of Chemical and Biomolecular Engineering, University of California, Berkeley, California 94720, United States; 5 Department of Chemistry & Biochemistry, University of California, La Jolla, San Diego, California 92093, United States; 6 Department of Nanoengineering, University of California, La Jolla, San Diego, California 92093, United States; 7 Department of Physics Materials Science Program, Colorado School of Mines, Golden, Colorado 80401, United States; 8 Advanced Energy Systems Graduate Program, Colorado School of Mines, Golden, Colorado 80401, United States; 9 Materials, Chemistry, and Computational Science Directorate, National Renewable Energy Laboratory, Golden, Colorado 80401, United States

## Abstract

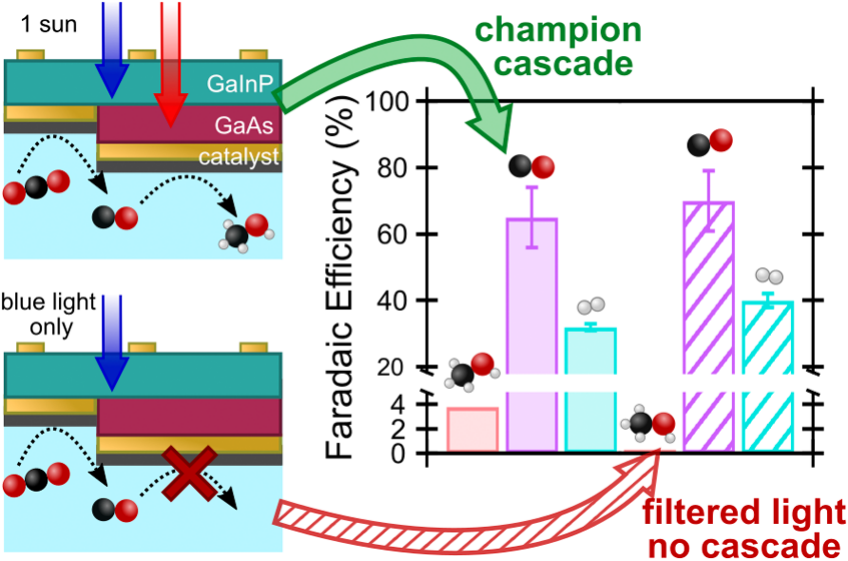

Biochemical networks use reaction cascades to selectively reduce CO_2_ using energy from sunlight, but can similar selectivity be achieved by applying a cascade approach to an engineered system? Here, we report the design and implementation of a two-step photoelectrochemical (PEC) cascade to a liquid solar fuel: reduction of CO_2_ to CO and subsequent reduction of CO to methanol. The potentials required to perform the reductions were generated using custom-made III–V-based three-terminal tandem (3TT) solar cells. Cobalt phthalocyanine immobilized on multiwalled carbon nanotubes (CoPc/MWCNT) catalyzed both reactions. Multiphysics simulations of electrolyte flow and nonilluminated electrochemical measurements were used to narrow the operating parameters for the CoPc/MWCNT 3TT photocathodes. The champion integrated photocathode produced methanol with 3.8 ± 0.4% Faradaic efficiency (FE), with tested photocathodes having 0.7–3.8% methanol FE. Products were quantified by nuclear magnetic resonance spectroscopy and gas chromatography. The current output of the tested photocathodes was highly stable, and methanol production continued over multiple experiments. The low methanol yield is attributed to insufficient CO flux to, and CO_2_ depletion at, the methanol-producing subcell when both contacts are active, which is supported by the observation that a control photoelectrode slightly outperformed the methanol production of the 3TT device. Methanol production ceased when the 3TT subcell driving CO reduction was deactivated, supporting the assignment of a cascade mechanism. The major factors resulting in low methanol FE by the CoPc/MWCNT 3TT photocathodes are insufficient CO_2_ depletion at the methanol-producing contact and uncertainty in operating potential selection using the 3TT design. Although the CoPc/MWCNT 3TT photocathode is not yet highly selective, this work develops the basic science principles underlying the PEC cascade, demonstrates the co-design of a 3TT-based photoelectrode to produce carbon-based fuels, and finally discusses routes for improving product yields with this concept, including CO_2_ supply optimization and alternative photoelectrode and catalyst materials.

## 1 Introduction

Photoelectrochemical (PEC) processes have produced the simplest solar fuel, hydrogen (H_2_), with solar-to-fuel efficiencies up to 30%.^1^ While there has been significant work toward scale-up and commercialization of PEC H_2_,^2,3^ existing liquid-based fuel infrastructure hinders commercial adoption of gaseous H_2_ fuel from any source.^4^ Alternatively, PEC carbon dioxide reduction (CO_2_R) is a method to produce drop-in liquid fuel replacements using the energy from sunlight.^5,6^ However, CO_2_R is more complex than H_2_ generation due to its range of possible products. CO production via PEC CO_2_R now approaches 20% solar-to-fuel efficiency,^7^ while PEC production of liquid products (e.g., methanol, ethanol, acetate) has not yet been demonstrated with high efficiency and selectivity.^8−11^ Methanol in particular is an attractive target for improving PEC CO_2_R efficiency, as it is the simplest “complex” reaction product (i.e., beyond CO or formate) and is widely used in chemical manufacturing and as a maritime fuel.^12−14^

In contrast to PEC CO_2_R, photosynthesis produces oxygenated C_2+_ products (e.g., glucose) with near-perfect selectivity.^15^ Notably, photosynthesis uses a series of enzymatic conversions at multiple sites to build up sugar molecules.^16^ Leveraging similar sequential chemical conversion steps without product isolation may be a route to improving CO_2_R selectivity and efficiency toward C_2+_ products.^17,18^ Control of intermediate transport between the reaction steps of such a system is required to match conversion rates for efficient operation;^19^ work on electrochemical CO_2_R cascades has shown that diffusional transport of intermediates is possible on the order of a typical electrochemical diffusion layer, ∼ 100 μm,^20,21^ and convective transport appears feasible over distances up to mm-scale.^22,23^ Similar cascades to improve selectivity of CO_2_R reactions have been demonstrated for combined (photo)electrochemical-photothermal systems.^24,25^

Design of a cascade process for (photo)electrochemical CO_2_R requires control of catalytic microenvironments to match the operating potential, current density, and catalyst to the desired reaction, as each reaction step has different thermodynamic requirements. Standard electrochemical reduction potentials (*E*^0^) for CO_2_R reactions range from −1.90 to 0.17 V_SHE_ (versus the standard hydrogen electrode),^26^ such as the two-step reduction of CO_2_ to methanol via a CO intermediate
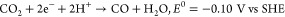
1
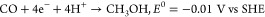
2where the sequential reduction half-reactions are assumed to occur with oxygen evolution as the oxidation half-reaction. To achieve the cascade production of methanol by this route, a CO-producing microenvironment must be coupled to a subsequent CO-consuming, methanol-producing microenvironment. In an electrochemical device, which does not directly utilize photons, this can be accomplished via bipotentiostatic measurements with independent control of two electrodes.^20,22^ In a PEC device, the semiconductor band gap and material quality set the potential and current flow is modified by changing the photoelectrode area in contact with the electrolyte.^27^ A catalyst anchored to a photoelectrode surface can reduce the overpotential needed to drive the reaction of interest.

Many photocathode architectures for CO_2_R have been demonstrated in the literature,^28^ both based on semiconductors used in photovoltaics (such as Si nanowires decorated with Cu nanoparticles)^29^ and on emerging materials (such as TiO_2_-coated Cu_2_O^30^ or CuInS_2_ thin films with CuFeO_2_ nanoparticles^31^). However, the need to drive not only both steps of the CO_2_R cascade, but also the kinetically slow oxygen evolution reaction, indicates that large photovoltages are practically required. Absorbing a larger portion of the solar spectrum by employing two semiconductors in a tandem configuration has been widely used in both PEC hydrogen generation^32−34^ and CO_2_R.^7^ Although tandems have enabled these demonstrations, series-connected devices cannot enable cascade reactions where two potentials are required; at the same time, the need to colocate catalytic microenvironments on a mm scale suggests the use of semiconductors integrated into a single device, rather than side-by-side illuminated photocathodes. We have previously shown using circuit modeling that a three-terminal tandem (3TT) photovoltaic device based on III–V semiconductors should be capable of providing control over the potential and current at two different colocated microenvironments of a CO_2_R cascade in a single device,^35^ although that model contained multiple simplifying assumptions. Other III–V-based devices have frequently been used as proof-of-concept platforms for PEC fuel-forming reactions.^32,33,36,37^

In addition to the design of a device that can provide separate potentials and current densities, such a photocathode must be coupled to appropriate catalysts to drive each step of the cascade. Cobalt pthalocyanine (CoPc) immobilized on multiwalled carbon nanotubes (MWCNT) has recently been reported to electrochemically produce methanol with Faradaic efficiency up to 50%,^38,39^ while also having a high FE toward CO at less negative potentials.^38,40^ In previous work, we found that CO was a dissociated intermediate in the pathway to methanol.^41^ CoPc anchored on various supports has long been used in PEC demonstrations with a single absorber and single catalyst site,^42^ including for CO_2_R.^40,43,44^ Inspired by the recent finding of CoPc’s dual functionality and previous reports of its use in PEC CO_2_R, we sought to use CoPc/MWCNT to catalyze both steps of cascade methanol production. Using the same catalyst in both microenvironments simplified construction of the 3TT photocathode and removed the possibility of cross-contamination between the catalyst sites (which could occur e.g. with two heterogenized metalorganic catalysts or with metallic catalysts plating during operation).^35^

**Figure 1:**
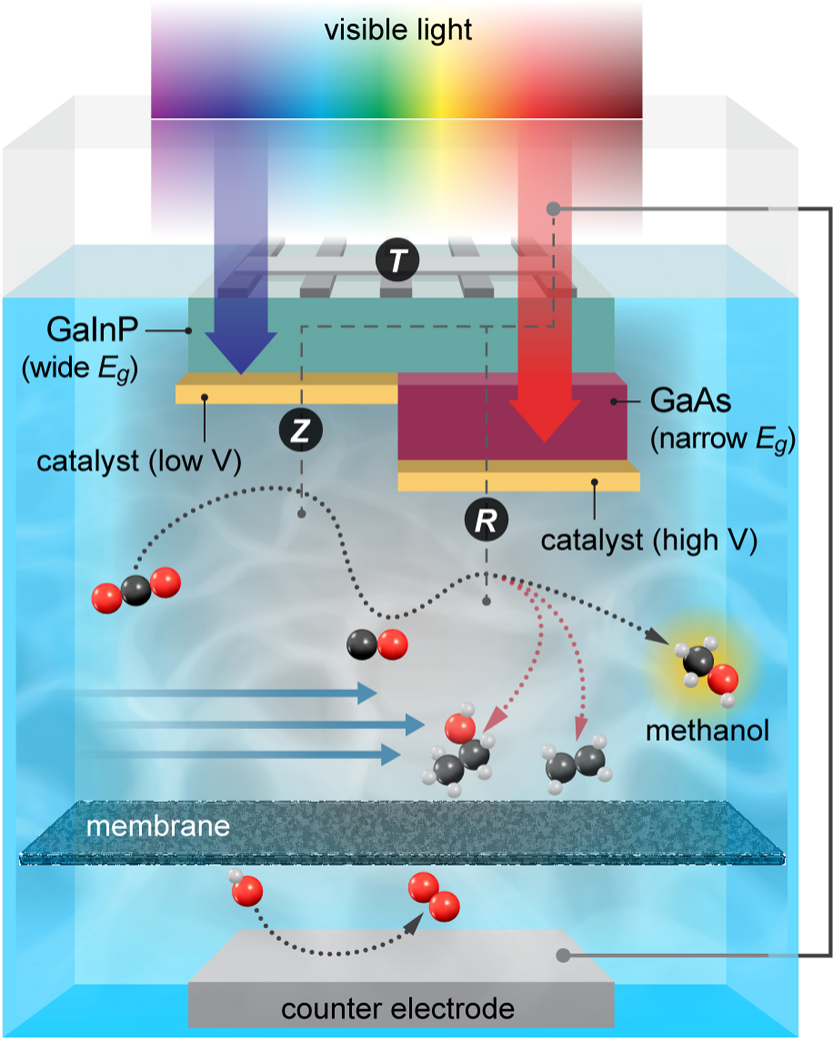
Schematic representation of a 3TT PEC device performing a cascade reaction where CO_2_ is reduced to CO at the Z contact and CO is subsequently reduced to methanol (rather than other CO_2_R products) at the R contact. Oxygen evolution occurs at the counter electrode, with a membrane between the electrodes to prevent product crossover. Illustration by Al Hicks, NREL.

Here we experimentally realize a proof-of-concept two-step PEC CO_2_R cascade with coupled microenvironments, using CO_2_ conversion to methanol as a model system. We demonstrate that 3TT photocathodes can provide the potentials and currents necessary to convert CO_2_ to methanol with CO as the intermediate (Figure 1) and develop an understanding of the fundamentals underlying the operation of such a device. III–V-based 3TT devices were modified from photovoltaic (PV) designs^45^ to enable two solution contacts with different potentials and thus, unique microenvironments. CoPc/MWCNT catalysts were used to drive both steps of the CO_2_R cascade reaction to methanol. 2D continuum modeling and nonilluminated electrochemical measurements were used to down-select electrolyte flow and catalyst potentials for PEC operation. The CoPc/MWCNT 3TT photocathodes produced methanol with a champion Faradaic efficiency of 3.8 ± 0.4% in a three-electrode configuration; no methanol was produced when the second reaction site was deactivated by blocking lower energy light. This initial demonstration of a PEC CO_2_R cascade with reactions occurring at distinct potentials suggests a promising path toward utilizing advanced photoelectrode designs to perform multistep PEC reactions, while demonstrating the need for light-absorbing and catalytic components of such systems to be co-designed rather than separately optimized.

## 2 Experimental Overview

We briefly overview the approach to synthesis and demonstration of the 3TT photocathode, with full details in the Supporting Information (**SI**). III–V-based 3TT photocathodes were grown by metalorganic vapor phase epitaxy (MOVPE) and fabricated into photovoltaic devices, which were tested using dry electrical methods to verify performance. The devices were then converted to photocathodes by the application of catalyst and protective layers. CoPc was dissolved in isopropanol with MWCNT to make a catalyst ink (hereafter, CoPc/MWCNT) that was applied to the photocathode terminals. Two approaches to modeling were used to reduce practical 3TT photocathode testing: a multiphysics continuum model of the electrolyte near the cathode surface simulating CO_2_ and CO transport via convection and diffusion, to determine electrolyte flow rate; and a non-light-active (“dark”) electrochemical device with only CoPc/MWCNT catalyst, to determine target 3TT operating potentials. The outputs of the multiphysics model and the practical electrochemical model set parameters for subsequent 3TT photocathode testing.

A three-electrode flow cell^41^ was used for both electrochemical and photoelectrochemical experiments. The anode and cathode chambers were separated by a Selemion membrane with a carbon paper counter electrode and Ag/AgCl reference electrode. The electrolyte was CO_2_-saturated 0.1 M KHCO_3_. For PEC experiments, CoPc/MWCNT 3TT photocathodes were illuminated from the T contact side (opposite to the contact with the electrolyte) using a Xe arc lamp with a water filter. A pyrometer was used to verify the light intensity for each PEC measurement. For experiments verifying the cascade mechanism, a short-pass light filter was used to block a portion of the lamp spectrum. Product quantification was performed with gas chromatography (CO and H_2_) and nuclear magnetic resonance spectroscopy (methanol).

## 3 Photocathode Design and Fabrication

A typical series-connected two-terminal tandem (2TT) photovoltaic device has two subcells and two terminals. Adding a third terminal to the tunnel junction between the subcells enables a three-terminal tandem (3TT) device; the terminals are denoted T (top), R (root), and Z (German *zusätzlich*, extra).^46^ For this PEC CO_2_R concept, illustrated in Figure 1, T is on the sun-facing side of the 3TT device, on one side of the GaInP subcell; Z is on the other, electrolyte side of the GaInP subcell (1.8 eV bandgap); and R is on the electrolyte side of the GaAs subcell (1.4 eV bandgap). Measuring from T to Z provides ∼1.4 V from the GaInP subcell, which should be sufficient to drive CO production (eq 1); we therefore expect the Z terminal to be the CO-producing microenvironment. Measuring from T to R provides ∼2.4 V from the two subcells connected in series (GaInP ∼ 1.4 V, GaAs ∼ 1 V), which should be sufficient to drive methanol production (eq 2); we therefore expect the R terminal to be the methanol-producing microenvironment. Tuning the areas of the terminals contacting the electrolyte tunes the electron fluxes for these reactions. The 3TT device structure used here was revised from photovoltaic designs to provide an areal ratio of Z to R of approximately 2:3 (Z = 0.367 cm^2^, R = 0.620 cm^2^ defined by the area where the Au contact is deposited).

**Figure 2:**
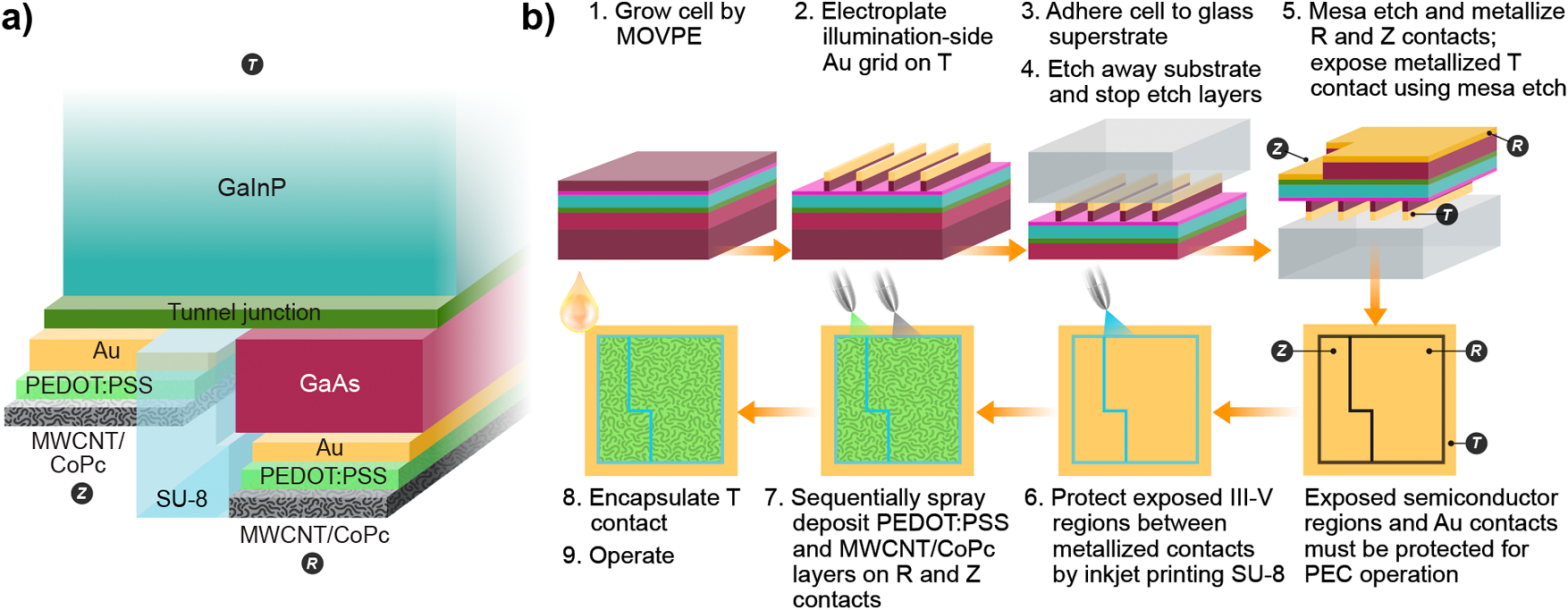
a) High-level schematic of the semiconductor, adhesion, and catalyst layers comprising the complete 3TT photocathode. b) General 3TT fabrication and catalyst layer deposition procedure (further details in the Supporting Information (**SI**)). Illustration by Al Hicks, NREL.

Figure 2 shows the 3TT photocathode assembly with catalyst integration and the overall fabrication process. III–V semiconductors were deposited with MOVPE (step 1 of Figure 2b) and fabricated into photocathodes using photolithography, metal deposition, and selective etching (details in Supporting Information (**SI**)). 3TT sample numbers (MVXXX or MUXXX) used throughout this work are assigned based on MOVPE deposition. Because the 3TT is mounted to a glass handle and the growth substrate is removed during processing (steps 3 and 4), this device is conceptually similar to “inverted” III–V 2TT growths (Figure S1),^47^ with different in situ annealing and a p-on-n orientation with respect to illumination.^48^ Gold contacts were initially electrodeposited on Z and R, but electron-beam evaporation improved device yield by reducing gold shunting between the two locations; samples produced with both methods are used in this study (step 5, detail in Figure S2). To protect the 3TT device from the aqueous PEC environment,^49−51^ any semiconductor not coated by gold was covered by inkjet-printing SU-8, an insulating epoxy (step 6). A spray-coated conductive poly(3,4-ethylenedioxythiophene) polystyrenesulfonate (PEDOT:PSS) layer was used to electrically connect and adhere the CoPc/MWCNT catalyst ink to the gold-coated Z and R terminals (step 7). The CoPc/MWCNT ink was spray-coated on to R and Z following PEDOT:PSS deposition. With contact to and protection of the T terminal, the CoPc/MWCNT 3TT photocathodes were ready for operation.

## 4 3TT Device Characterization

**Figure 3:**
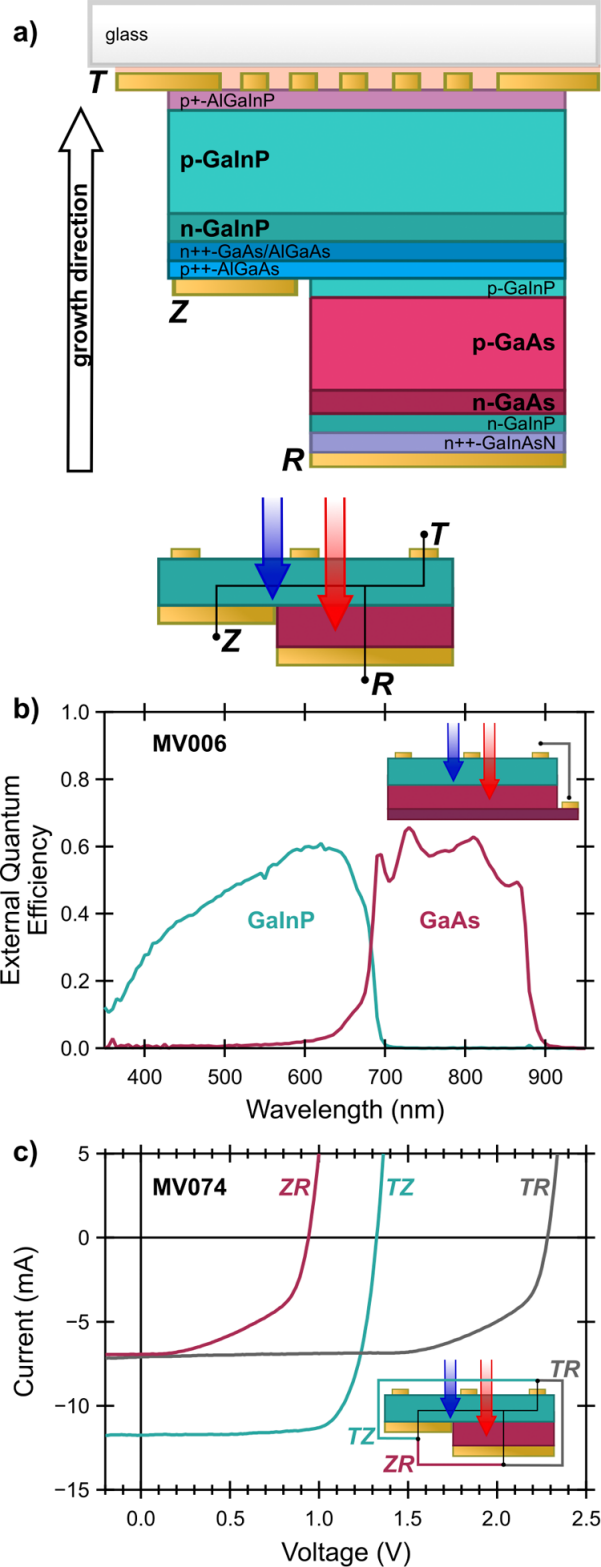
a) Detailed schematic (top) of III–V layers for the 3TT devices, including the direction of layer growth and simplified schematic (bottom) of the 3TT device showing electrical pathways. The schematic is not to scale and not all layers are shown, see Supporting Information (**SI**). b) External quantum efficiency of a 3TT growth fabricated as a test structure (not removed from the growth substrate, see inset and Figure S1). c) *I–V* curves for a representative 3TT device under AM 1.5G illumination with inset schematic showing measurement configurations.

The high-level structure of the 3TT III–V device is shown in Figure 3a (complete detail in Table S1). To confirm the quality of the GaInP and GaAs subcells, the quantum efficiency of a test structure, fabricated from the same MOVPE growths as 3TT photocathodes, was measured using external light bias to separate the subcell responses (Figure 3b).^52^ The test structure was fabricated upright without a glass handle and no Z contact to simplify analysis. Both subcells have the expected, reasonable external quantum efficiency across their wavelength ranges, consistent with no antireflection coating. Further details and a discussion of general considerations for 3TT device characterization can be found in the **SI** (Figure S3).

After fabrication (through step 6, Figure 2b), dry photovoltaic current–voltage (*I*-*V*) characteristics of the 3TT devices were measured between pairs of terminals: TR measures the series-connected tandem; TZ measures the GaInP subcell only; ZR measures the GaAs subcell. Figure 3c shows *I*-*V* characteristics of a representative 3TT device (see Table S2 for full details for all devices, including sample numbers). For devices with evaporated gold contacts, TR open-circuit voltage (*V*_OC_^TR^) was 2.26–2.30 V, equivalent to the summed TZ and ZR *V*_OC_, where *V*_OC_^TZ^ was 1.31–1.34 V and *V*_OC_^ZR^ was 0.94–0.99 V; these values are close to the expected voltages based on the band gaps of the semiconductors. In the TR configuration, the current through the series-connected tandem is limited by the GaAs subcell to a short-circuit current (*I*_SC_^TR^) of 6.7–7.1 mA. Fill factors (*FF*) varied from device to device, largely due to shunting between Z and R, but were >65% for *FF*_TR_ and *FF*_TZ_ in devices with evaporated contacts. The overall dry performance of MU845, the device with electroplated gold contacts, was slightly lower, with *FF*_TR_ = 53% and *FF*_TZ_ = 55%. As noted above, electroplating was found to result in more gold bridging Z and R, leading to losses due to shunting. The dry characterization of the 3TT devices shows they will produce sufficient potential and current to drive the desired CO_2_R cascade reaction.

## 5 CO_2_R Cascade Model Systems

To reduce manual exploration of the operating parameter space for the 3TT photocathodes, we designed two models. The first model addressed electrolyte flow rate, which determines CO_2_ transport to Z, the CO-producer, and subsequent CO transport to R, the methanol-producer. To simulate CO and CO_2_ transport via diffusion and convection, a 2D continuum multiphysics model of the electrolyte near the cathode surface was developed, the details of which can be found in the **SI**Figures S4–S6. For our system, the model predicts maximized methanol generation from CO at an electrolyte inlet flow rate of ∼8.5 cm/min, which balances mass transport and chemical conversion rates and is consistent with prior modeling and experiments.^41^ However, methanol production is predicted to be more sensitive to flow rate over the 3TT photocathodes than in previous work due to the 2 μm height of the protective SU-8 coating between the Z and R terminals. The flow rate of 8.5 cm/min was used for all (photo)electrochemical experiments.

**Figure 4:**
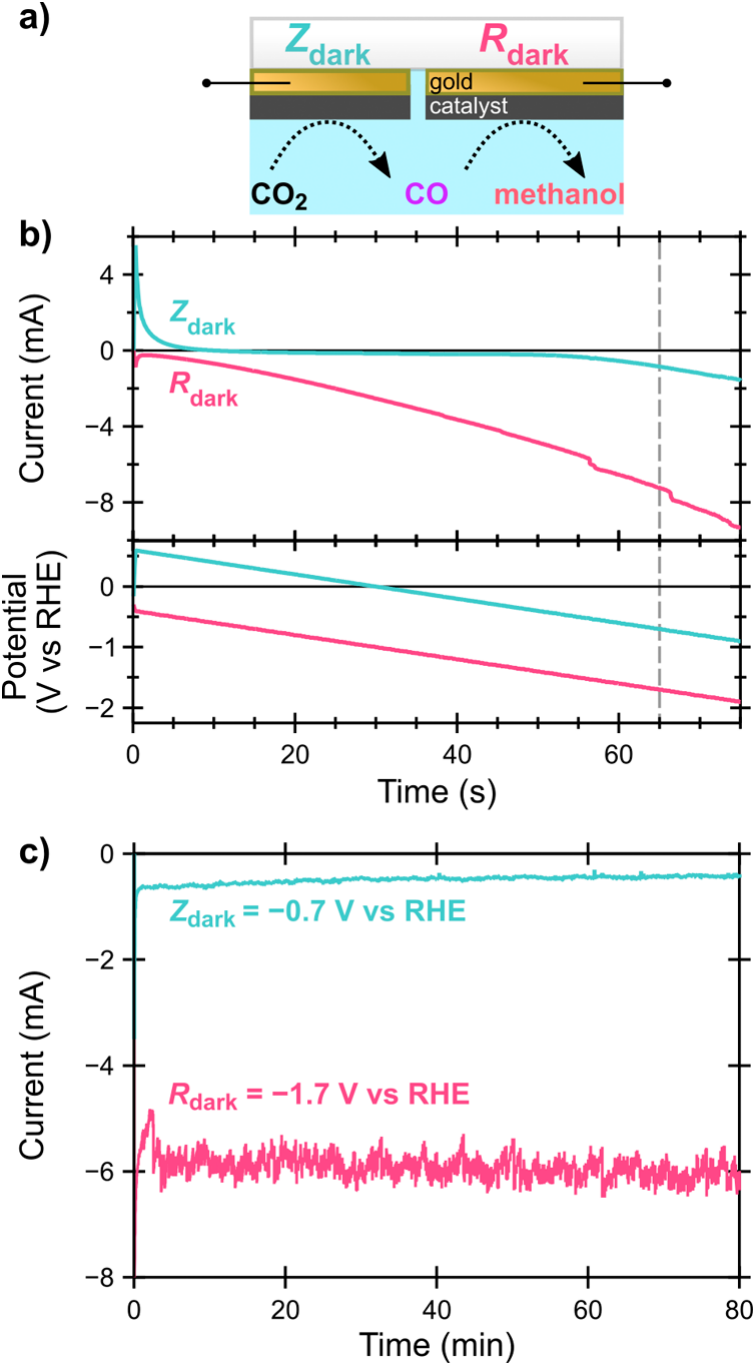
a) Schematic of the dark electrochemical device operated by a bipotentiostat (electrical connections illustrated by black lines) in order to simulate the 3TT photocathode. b) Linear sweep voltammetry of Z_dark_ (area: 0.56 cm^2^) and R_dark_ (0.36 cm^2^) with current and potential versus time; the vertical gray dashed line indicates the time where R_dark_ = −1.7 V vs RHE and Z_dark_ = −0.7 V vs RHE, 1 V less reducing. c) CA measurement of the same electrochemical device with R_dark_ = −1.7 V vs RHE. For electrochemical measurements, a CoPc/MWCNT working electrode, Ag/AgCl reference electrode, and carbon counter electrode were operated in 0.1 M KHCO_3_ aqueous electrolyte saturated with CO_2_. The working and counter electrodes were separated by a Selemion membrane. These data were not compensated for solution resistance.

To determine target operating potentials for the microenvironments for methanol generation, a non-light-active (“dark”) electrochemical device was operated as the second, experimental model in the two-compartment flow cell that would later be used for PEC testing. Two closely spaced, electrically insulated gold electrodes were fabricated with the same catalyst layers and similar areas as the CO-producing and methanol-producing terminals. These dark electrodes are referred to as Z_dark_ (the CO producer) and R_dark_ (the methanol producer) and are illustrated in Figure 4a (photograph in Figure S7). Z_dark_ and R_dark_ potentials were constrained to a 1 V difference via a bipotentiostat to mimic the expected potentials of Z and R under AM 1.5G illumination of the 3TT photocathode. Linear sweep voltammetry in the three-electrode flow cell with CO_2_-saturated 0.1 M KHCO_3_ reveals that there is *no* condition within the measured potential window where the currents produced by Z_dark_ and R_dark_ have the 1:2 ratio required by the stoichiometry of our methanol-producing cascade (eqs 1 and 2). Negligible current is passed by Z_dark_ until R_dark_ reaches a more negative potential than −1.7 V versus the reversible hydrogen electrode (RHE, Figure 4b), indicating that little CO can be produced at Z_dark_ until that point. Constant potential, chronoamperometry (CA) measurements were also performed with R_dark_ poised at −1.7, – 1.8, and −1.9 V vs RHE. The maximum methanol FE (15 ± 4% over three experiments) occurred when R_dark_ = −1.7 V vs RHE (Z = −0.7 V vs RHE), but the current at Z_dark_ was much less than half of R_dark_ (Figure 4c). At the more reducing potentials (R_dark_ = −1.8 or −1.9 V vs RHE), H_2_ FE was high, as expected for CoPc/MWCNT.^38,39,41,53^ These potentials are consistent with our prior CoPc/MWCNT-catalyzed methanol work^41^ after correcting for solution resistance (see Supporting Information (**SI**)).

The discrepancy between the methanol production for the dark electrodes (15 ± 4% FE) and our prior work (36 ± 3% FE on a 1 cm^2^ electrode)^41^ can be explained by the dependence of CoPc/MWCNT selectivity toward methanol on the supply of CO in addition to the depletion of CO_2_, since CO_2_ outcompetes CO for active sites.^54,55^ In our cascade, due to the 1 V difference between Z and R set by the design of our 3TT photocathodes, Z_dark_ cannot reach highly negative potentials with the optimal potential of R_dark_ at −1.7 V vs RHE. Since Z_dark_ potential and current were low, little CO was produced by that electrode and there was insufficient CO_2_ depletion at R_dark_ to enable methanol production from CO by the CoPc/MWCNT catalyst. Although FE was low, methanol was produced by the dark electrochemical cascade model, indicating that a solar-driven PEC cascade demonstration should be feasible within the constraints of the 3TT photocathode design.

## 6 CO_2_R Cascade on CoPc/MWCNT 3TT Photocathodes

With electrolyte flow (8.5 cm/min) and R target operating potential (hereafter *V*_op_, – 1.7 V vs RHE) determined using the model systems, the full CO_2_R cascade could be explored using the CoPc/MWCNT 3TT photocathodes (photograph of device in Figure S8). The 3TT device geometry prevents the direct measurement of the potentials at the Z and R terminals during PEC operation; thus, the necessary T terminal operating potential (the working electrode potential) was determined separately for each photocathode and used to estimate the R and Z contact potentials. The potential at which to set the T contact for methanol-producing CA experiments (*V*_CA_) was determined using the following equation:
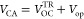
3

Using *V*_OC_^TR^ should ensure that the R contact is sufficiently reducing to convert CO to methanol; as described above, the Z contact will be about 1 V less reducing than the R contact, sufficient to convert CO_2_ to CO.

**Figure 5:**
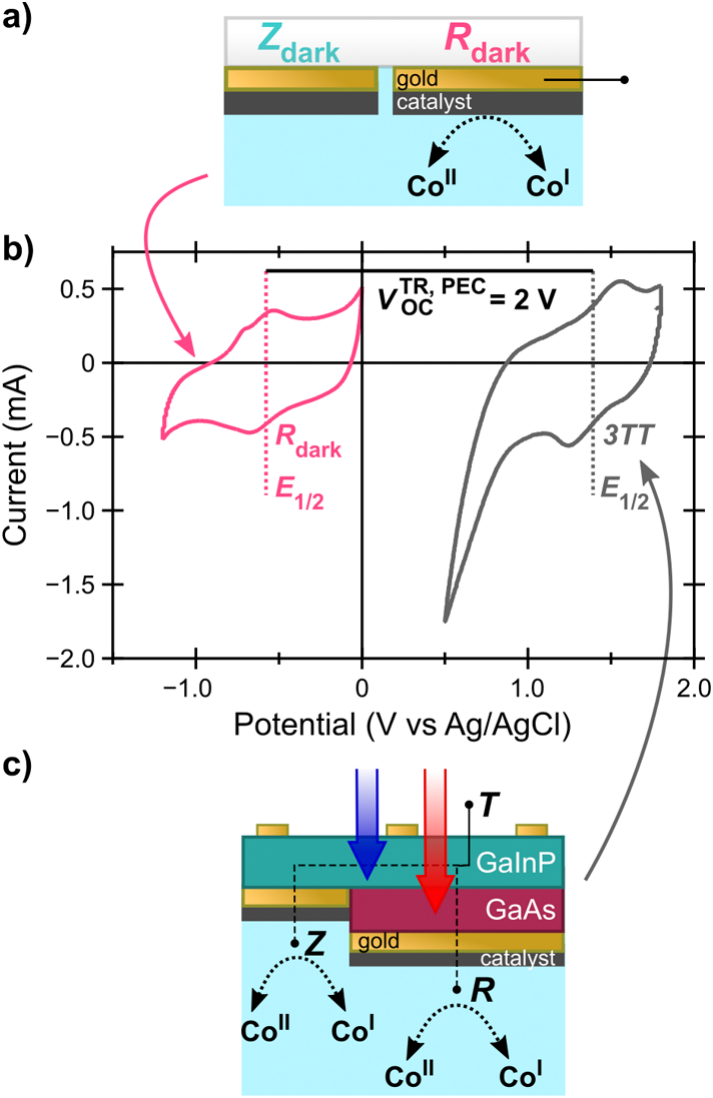
Determination of *V*_CA_ potentials for CoPc/MWCNT 3TT photocathode demonstrations. a) Schematic of dark electrochemical model device, where only the R_dark_ contact is operated to determine the Co^2+^/Co^1+^*E*_1/2_. b) Current–voltage characteristics of electrochemical model (pink) and 3TT (gray) CoPc/MWCNT layers. c) Schematic of CoPc/MWCNT 3TT photocathode operating to determine *E*_1/2._ Electrochemical and PEC measurements were performed in the same three-electrode flow cell. All other conditions were the same as for other experiments. These data were not compensated for solution resistance.

However, dry *V*_OC_^TR^ varied between 3TT devices (Table S2), and some resistive drop across the PEDOT:PSS adhesion layers is expected. We therefore attempted to derive a value from PEC measurements for effective R contact photovoltage (*V*_OC_^TR, PEC^) for use in eq 3 rather than *V*_OC_^TR^. Determination of *V*_OC_^TR, PEC^ from a 3TT photocathode is convoluted because the geometry of the device only allows for the potential difference to be measured between the T contact and the counter electrode, rather than measurement of the voltage at the R (or Z) contact. *V*_OC_^TR, PEC^was estimated by comparing the CoPc (Co^II^/Co^I^) or methyl viologen (MV^2+^/MV^+^)^56−58^ reduction half-wave potential (*E*_1/2_) on an illuminated 3TT photocathode to the same reduction on a dark electrode (illustrated in Figure 5). The difference in *E*_1/2_ was taken to be *V*_OC_^TR, PEC^, with inclusion of overpotentials and Fermi level pinning effects.^59^ However, we caution that the difference in dark and light *E*_1/2_ only *approximates V*_OC_^TR, PEC^ because this value is determined from *E*_1/2_ of the Z and R contacts convoluted together, measured through the T contact. Further detail of *V*_OC_^TR, PEC^ determination can be found in the **SI** (Table S4).

**Figure 6:**
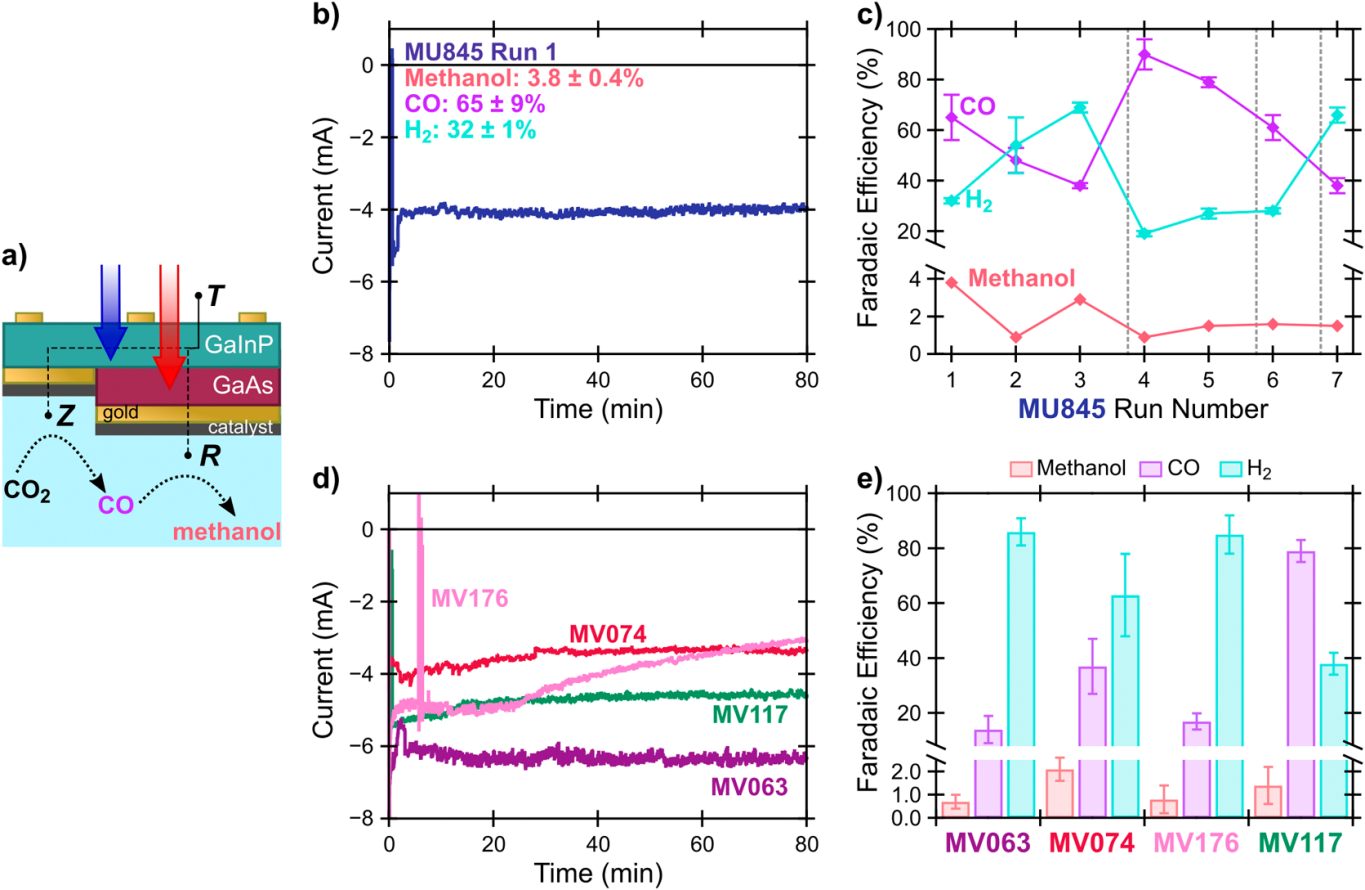
Demonstration of 3TT photocathode cascade. a) Schematic of 3TT operation under simulated AM 1.5G illumination (see Supporting Information (**SI**)). b) CA measurement of pristine 3TT photocathode MU845 producing methanol at 3.8 ± 0.4% FE, held at +0.3 V vs RHE. c) Comparison of FEs toward methanol, CO, and H_2_ for repeated operation of MU845 (see Table S4). Dashed gray lines indicate regeneration of the catalyst by drop-casting. d) CA measurement of pristine 3TT photocathodes MV063, MV074, MV117, and MV176. e) FEs of the four CoPc/MWCNT 3TT photocathodes toward methanol, CO, and H_2_. PEC measurements used the same conditions as electrochemical measurements, with the 3TT photocathode as the working electrode. The reference electrode was Ag/AgCl and the counter electrode was carbon, operated in 0.1 M KHCO_3_ aqueous electrolyte saturated with CO_2_; the working and counter electrodes were separated by a Selemion membrane. These data were not compensated for solution resistance.

The schematic in Figure 6a illustrates testing of 3TT photocathodes performing cascade conversion of CO_2_ to methanol under nominal AM 1.5G illumination (Xe arc lamp with water filter, see SI and Figure S9) in the two-compartment three-electrode flow cell in CO_2_-saturated 0.1 M KHCO_3_. The champion CA measurement on a CoPc/MWCNT 3TT photocathode (MU845) is shown in Figure 6b, which produced methanol with 3.8 ± 0.4% FE. The current from the photocathode remained steady across the 80 min measurement. MU845 also produced methanol on subsequent CA measurements, although at lower FE even with regeneration of the catalyst ink via careful drop casting of new material (Figure S10); repeated regeneration of a single 3TT photocathode yielded methanol FE between 0.9 and 2.9% across seven experiments (Figure 6c). Four other CoPc/MWCNT 3TT photocathodes produced some amount of methanol; the CAs for these devices are shown in Figure 6d and the product yields are shown in Figure 6e. Thus, we have successfully demonstrated PEC production of a liquid solar fuel using CoPc/MWCNT 3TT photocathodes.

Although all five CoPc/MWCNT 3TT photocathodes successfully produced methanol, MU845 outperformed the others in terms of FE despite having poorer dry *I–V* characteristics. In fact, we attribute the methanol production performance of MU845 to the smaller *V*_OC_^TZ^, which led to *V*_CA_ being more reducing for this device than the other photocathodes, likely making the actual potentials at Z and R more reducing overall. This conclusion is supported by the overall high production of CO compared to H_2_ by MU845, relative to the other 3TT photocathodes.

**Figure 7:**
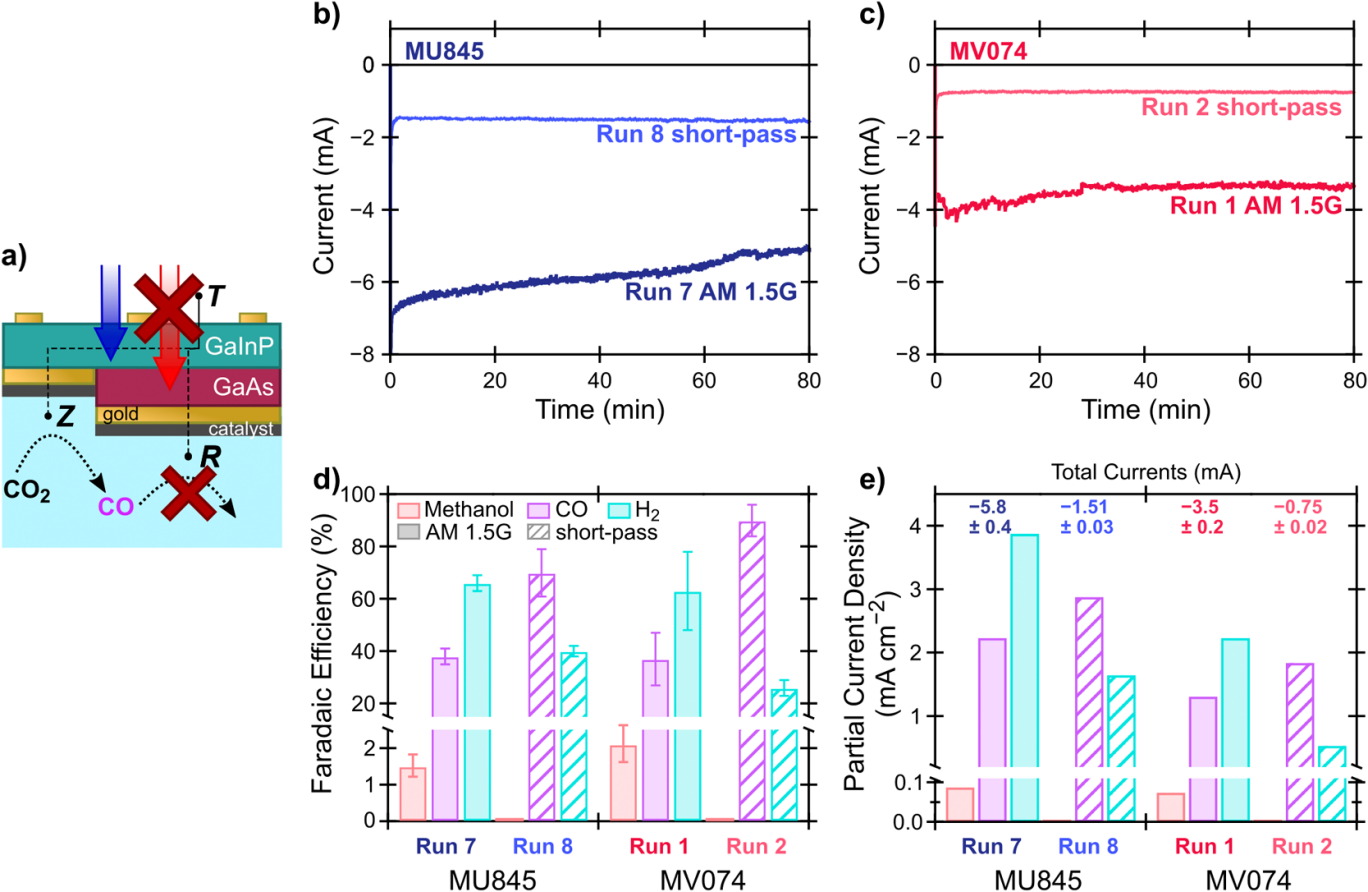
a) Schematic of 3TT operation under a 650 nm short pass light filter, where R does not contribute to current generation nor produce a voltage because no light with a wavelength greater than 650 nm (represented by the red arrow) reaches the GaAs subcell. b) CA measurement of MU845 under AM 1.5G illumination (dark blue trace) and under short-pass filtered illumination (light blue trace). c) CA measurement of MV074 under AM 1.5G illumination (dark red trace) and under short-pass filtered illumination (light red trace). d) FEs under AM 1.5G (dark labels) and short-pass filtered (light labels) illumination on MU845 and MV074. e) Partial current densities toward products normalized based on both Z and R being active under AM 1.5G (0.987 cm^2^ active area in contact with electrolyte) and only Z being active with the short-pass filter in place (0.367 cm^2^ active in contact with electrolyte). Total currents are given at the top of f); note that due to normalization, the partial current densities for the experiments where R is deactivated do not sum to the total current. These data were not compensated for solution resistance.

In this cascade PEC CO_2_R concept, methanol should be produced from CO (eq 2) at R, which is poised at the more negative potential (Figure 1). However, as described above, the CoPc/MWCNT catalyst is present at *both* R, the methanol-producing terminal, and Z, the CO-producing terminal. Although this eliminates concerns of catalyst cross-contamination, it does raise the question of where methanol is actually produced. To address this, we experimentally deactivated the methanol producer, by using a 650 nm short-pass filter to block light below the bandgap of the GaInP subcell, functionally zeroing the current passed at R, which lies on the GaAs subcell (Figure 7a). As shown in Figure 7b and c, use of the short-pass filter substantially reduced the current output from MU845 and MV074; the zeroing of current from R was directly confirmed by dry *I–V* measurements (Figure S11). With no current passing at R to the CoPc/MWCNT catalyst, we expect to see no methanol produced if the cascade mechanism is occurring. Figure 7d shows that in two replicate experiments, R deactivation results in the production of methanol falling to undetectable levels (<0.05 mM by NMR, corresponding to <0.07% FE). We conclude that the R terminal is in fact the methanol producer.

As expected when the amount of light absorbed by the 3TT device is limited by the short-pass light filter, the current at R is effectively zeroed, no methanol is produced and FE toward CO increases, as the CoPc/MWCNT catalyst potential is limited to the voltage generated at Z. While this is compelling evidence for a cascade mechanism, CO produced by Z cannot be chemically distinguished from undesired CO produced at R as a side product to methanol. Thus, it is possible that under AM 1.5 G illumination CO produced at R is then directly reused at R to produce methanol, rather than methanol production from CO that flows from Z to R. Such a scenario would also result in no methanol production when the short-pass light filter is in place. However, we analyzed the partial current densities toward each product under simulated AM 1.5G illumination and with the short-pass filter, based on the 0.987 cm^2^ area of the full 3TT photocathode and the 0.367 cm^2^ area of Z, respectively (Figure 7e). Both the partial current density analysis and FE show that when the 3TT photocathode is fully illuminated, there is a higher production of H_2_, consistent with the very negative expected potential at R. Normalization of the currents reveals that the partial current density toward CO *increases* when only Z is active under filtered illumination, strongly indicating that some CO produced at Z is consumed at R to make methanol under AM 1.5G.

Because the GaInP and GaAs subcells are connected in series, analogous deactivation of the Z contact with a long-pass light filter would shut off current flow through the entire device, deactivating the R contact at the same time. However, a 2TT photoelectrode presents the same voltage at the R contact while eliminating the Z contact from the device (Figure S1). Therefore, control experiments with a CoPc/MWCNT 2TT photocathode were performed to mimic an active R contact with no Z contact. CA of the 2TT control at an equivalent applied potential to the 3TT devices (*V*_CA_, eq 3) resulted in 5 ± 2% FE toward methanol (see Supporting Information (**SI**)) from direct sequential reduction of CO_2_ to CO and then to methanol. The photovoltage of the 2TT can more accurately be estimated using eq 3 than the photovoltage of the 3TTs as it only has one electrolyte contact, which may have contributed to the higher methanol production with this photocathode compared to the champion 3TT (3.8 ± 0.4% FE); the larger active area of the 2TT compared to the R contact (1.21 cm^2^ vs 0.620 cm^2^) likely also contributed to higher methanol production on the 2TT.

While the five tested CoPc/MWCNT 3TT photocathodes produced methanol, they were outperformed by the 2TT control photocathode (FE = 5.3 ± 2.2%) and the dark electrochemical model (FE = 15 ± 4%). We attribute this discrepancy to the unoptimized voltages at the Z and R contacts of the 3TT and resistive losses in the CoPc/MWCNT layer (see eq 3 discussion). There is also pronounced competition between CO and CO_2_ binding at the CO-consuming R contact,^54,55^ reducing the efficiency of both the illuminated and dark systems (where methanol FE falls short of our previously demonstrated 36 ± 3%).^41^ Though the overall FEs of the 3TT devices are low, the history of advances in PEC solar fuel demonstrations illustrates that improved performance is likely highly accessible (consider the ∼0.4% solar-to-hydrogen efficiency on polycrystalline TiO_2_ demonstrated by Fujishima et al. compared to modern records)^60^ through the co-design of catalysts, semiconductor architecture, and mass transport. Our prior circuit modeling work^35^ calculated higher efficiencies for 3TT photocathodes than obtained here; although there are multiple differences between that model and the realized device structure, we anticipate that changes to the 3TT design based on the understanding gained in this study will afford improved performance in the future. A better understanding of the behavior of a 3TT photocathode in contact with electrolyte would also reduce uncertainty in determining *V*_CA_ and thereby improve 3TT methanol FE. This would likely enable higher methanol FE using the cascade than using a 2TT photocathode, especially considering that at present FEs are low on both types of photocathodes.

## 7 Conclusions and Design of Future 3TT Photocathode Systems

In this study, a two-step PEC CO_2_R cascade was catalyzed by CoPc/MWCNT on a III–V-based 3TT photocathode and achieved 3.8 ± 0.4% FE toward methanol, demonstrating a successful proof-of-concept for this device structure. Custom III–V-based devices were designed and grown to act as the photocathodes, and CoPc/MWCNT was used as the catalyst ink. Multiphysics simulations were used to model CO_2_ and CO transport and identified ∼8.5 cm/min as the target flow rate for cascade experiments. A non-light-active electrochemical device mimicking the 3TT device was used to explore cascade reactions within the potential constraints of the photocathode. Experimental demonstrations of CoPC/MWCNT 3TT photocathodes operating a PEC CO_2_R cascade produced methanol across multiple devices and repeated operation of a single device. Finally, a short-pass filter was used to demonstrate that the cascade mechanism is likely driving methanol production.

The need to simultaneously control multiple components (e.g., semiconductor device structure and geometry, electrolyte flow, catalyst choice and deposition method, microenvironment potential) creates a wide co-design space for 3TT-based photocathodes that requires much more exploration. This proof-of-concept study demonstrates the need for co-design, rather than independent optimization of components, and helps identify areas for future development to improve the efficiency of the cascade PEC CO_2_R. Tuning contact areas and geometries would increase the area of the R contact where CO_2_ is depleted, enhancing methanol production. Changing the III–V semiconductors (and thus bandgaps)^61^ used in the subcells of the 3TT would tune the cascade microenvironment potentials, which can also be better controlled by using more conductive adhesion layers to drive catalysts without potential loss. The development of 3TT photovoltaic devices based on alternative materials systems, such as Si/perovskite tandems,^62,63^ would enable an even broader range of photocathode characteristics, once devices with the appropriate configurations^46^ have been designed. CoPc/MWCNT was the only catalyst investigated in this study; alternative catalysts that more efficiently produce CO (at the Z terminal) or selectively consume CO (at the R terminal) would improve overall FE toward liquid fuel production, while adding complexity in 3TT photocathode design. For CO_2_ reduction to CO, a Re- or Ru-based molecular catalyst would be highly active and selective.^64,65^ A catalyst that is highly selective for reduction of CO rather than CO_2_R would be needed on the R terminal; possible alternatives could include highly strained CoPc that is more selective toward methanol^39^ or oxide-derived copper that can convert CO into multicarbon products.^66,67^ Although gas diffusion layers (GDLs) are not currently compatible with photoelectrodes, GDL CO_2_R microenvironments have been shown to be highly tunable,^68^ which may also provide a route for 3TT catalyst development. With these improvements to the 3TT photocathode, cascade PEC processes may represent a new platform to produce carbon-based solar fuels with high selectivity. This work demonstrates that co-design of such a system is possible and lays the groundwork for future research in this area.

## Data Availability

The raw data underlying this study will be uploaded to a public repository upon publication.

## Supporting Information Available

Detailed experimental methods, 3TT device design, synthesis and processing, and dry device characterization; continuum model for (photo)electrochemical operating conditions; catalyst materials and layer deposition; PEC experimental setup including illumination, determination of T contact operating potentials for CA, product detection, regeneration of catalyst, and light filter experiments (PDF)
