# Supplementary material for: Realization of a Photoelectrochemical Cascade for the Generation of Methanol: A Liquid Solar Fuel
Source: Energy Fuels. 2024 Dec 23;39(8):4019–29. doi: 10.1021/acs.energyfuels.4c04779 (PMC11874017; doi:10.1021/acs.energyfuels.4c04779)
Supplement: Supplementary file 1 — ef4c04779_si_001.pdf [file ef4c04779_si_001.pdf]

Realization of a Photoelectrochemical Cascade for the Generation of Methanol: A Liquid Solar Fuel

Thomas Chan,[1,5,6,#] *Calton J. Kong,*[1,3,#] *Grace A. Rome,*[7,9,#] *Darci Collins,*[8,9] *Alex J. King,*[1,4] *Rajiv Ramanujam Prabhakar,*[1] *Sarah A. Collins,*[9] *Michelle S. Young,*[9] *Mickey J. Wilson,*[9] *Myles A. Steiner,*[9] *Adele C. Tamboli,*[7,9] *Emily L. Warren,*[9*] *Clifford P. Kubiak,*[5,6*] *Joel W. Ager,*[1,2,3*] *Ann L. Greenaway*[9*]

[1]Chemical Sciences Division, [2]Materials Sciences Division, Lawrence Berkeley National Laboratory, Berkeley CA 94720, United States

[3]Department of Materials Science and Engineering, [4]Department of Chemical and Biomolecular Engineering, University of California, Berkeley CA 94720, United States

[5]Department of Chemistry & Biochemistry, [6]Department of Nanoengineering, University of California, San Diego, La Jolla CA 92093, United States

[7]Department of Physics Materials Science Program, [8]Advanced Energy Systems Graduate Program, Colorado School of Mines, Golden CO 80401, United States

[9]Materials, Chemistry, and Computational Science Directorate, National Renewable Energy Laboratory, Golden CO 80401, United States

[#]Authors contributed equally

***Corresponding Authors:** Emily L. Warren (emily.warren@nrel.gov)**,** Clifford P. Kubiak (ckubiak@ucsd.edu), Joel W. Ager (jwager@lbl.gov), Ann L. Greenaway (ann.greenaway@nrel.gov)

Table of Contents

THREE-TERMINAL TANDEM (3TT) DEVICES.......... 3

DEVICE DESIGN 3
SYNTHESIS AND PROCESSING.......... 4
DRY DEVICE CHARACTERIZATION.......... 6

CONTINUUM MODEL FOR (PHOTO)ELECTROCHEMICAL OPERATING CONDITIONS.......... 7

CO_2R COPC/MWCNT CATALYST.......... 10

CATALYST MATERIALS.......... 10
CATALYST LAYER DEPOSITION.......... 11

CASCADE (PHOTO)ELECTROCHEMISTRY.......... 12

EXPERIMENTAL SETUP.......... 12
ILLUMINATION.......... 13
DETERMINATION OF T CONTACT OPERATING POTENTIALS FOR CHRONOAMPEROMETRY.......... 13
PRODUCT DETECTION.......... 14
REGENERATION OF COPC CATALYST.......... 15
LIGHT FILTER EXPERIMENTS.......... 16

REFERENCES.......... 17

Three-Terminal Tandem (3TT) Devices

Device Design

A typical two-terminal tandem (2TT) is a photovoltaic device with two series-connected subcells that can source one voltage between its two terminals. 3TTs are photovoltaic devices with two series-connected subcells and *three* terminals, where different voltages can be sourced between binary combinations of the three terminals.[1] As discussed in the main text, the III-V-based 3TT devices constructed in this work are conceptually similar to “inverted” 2TT growths but with two major differences, which are illustrated in **Figure S1**. Using the nomenclature established by Warren et al.,[1] the 3TT photocathode devices have GaInP/s/GaAs(p/n) structure.

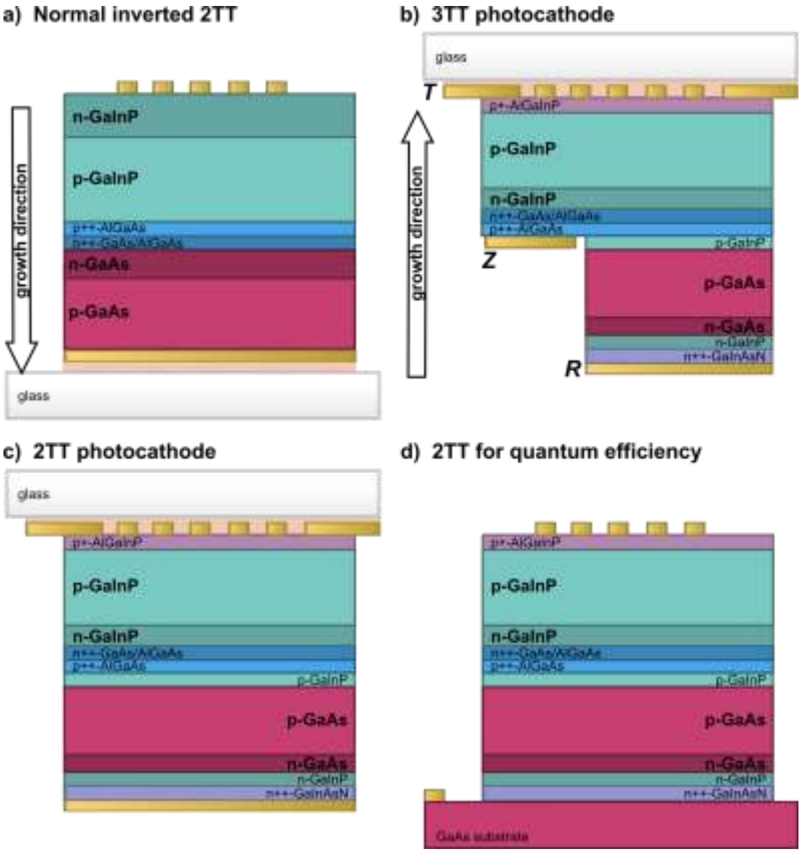

Figure S1: Device layers and growth direction for a) a standard inverted 2TT III-V-based photovoltaic device, b) a 3TT photocathode device, c) a 2TT from the same growth recipe as the 3TT photocathodes fabricated as a 2TT for PEC operation, and d) a 2TT from the same growth recipe, fabricated as an on-substrate device for quantum efficiency measurements. Note that not all layers are shown, see **Table S1**.

First, while a normal inverted 2TT (**Figure S1a**) has the GaInP grown first, followed by the GaAs, for the 3TT (**Figure S1b**) the GaInP top cell is grown after the GaAs bottom cell. The lower band gap material, GaAs, is grown first in the 3TT such that it is at the bottom of the tandem, but is grown second in a 2TT to achieve the same illumination order; the growth order of the GaInP subcell is similarly swapped between the two device types. This swap changes the in situ annealing conditions of both cells relative to standard inverted MOVPE growths, requiring re-optimization

of the growth conditions to form high quality interfaces and create the desired doping and annealing profiles. Second, because CO_2R requires electrons and the 3TT must operate as a photocathode, the cell has a p-on-n orientation with respect to illumination[2] where most III-V photovoltaic devices have n-on-p structures to take advantage of the higher electron mobility in n-type current-spreading layers.[3] Changing the order of subcell growth and the device polarity also affects each interface: for instance, the GaAs subcell is grown on the n++ layer of the tunnel junction for the 2TT, but for the 3TT the GaInP is grown on the n++ layer of the tunnel junction, potentially changing the number of defects at that interface. **Figure S1c** shows the structure of a 2TT photocathode control and **Figure S1d** shows the layers of a 2TT for quantum efficiency measurements; both of these were fabricated from identical MOVPE growths to the 3TT samples (step 1 of **Figure 3b**).

Synthesis and Processing

Samples were grown in a custom-built atmospheric-pressure metalorganic vapor phase epitaxy (MOVPE) reactor on (100)-oriented n-type GaAs substrates, miscut 2° toward (111)B. The source materials for the growth included triethylgallium, trimethylgallium, trimethylindium, and trimethylaluminum for the group-III sources; phosphine and arsine for the group-V sources; dimethylhydrazine for nitrogen; and dilute hydrogen selenide, diethylzinc, disilane, and carbon tetrachloride for the dopants. All gasses were mixed with a purified hydrogen carrier gas at a flow rate of 6 L/min. The substrates were cleaved to be 1.7 cm × 3.4 cm rectangles, etched in 2:1:10 (by volume) $NH_4OH:H_2O_2:H_2O$ solution, rinsed with deionized water, and loaded into the MOVPE reactor under nitrogen. The sample was ramped to 700°C with an arsine overpressure, and then held at 700°C under arsine for 10 minutes to deoxidize the surface. Following growth, the sample was cooled to room-temperature under an arsine overpressure. **Table S1** details the growth layers.

Table S1: List of MOVPE-deposited III-V layers in a 3TT, in the order of deposition and the function of each layer.

Layer	Purpose
GaAs	purchased substrate
GaAs	seed layer
GaAlAs	
GaInP	stop etch
n++ GaInAsN	contact layer (**R contact**)
n++ GaAs	
n GaInP	back surface field for GaAs cell
n GaAs (Se dopant)	GaAs n-type
p GaAs (Zn dopant)	GaAs p-type
p GaInP	window (front passivation) for GaAs cell
p++ AlGaAs	p++ Tunnel Junction / **Z contact** layer
n++ GaAs/AlGaAs	n++ Tunnel Junction
n+ AlInP	back surface field for GaInP cell
n GaInP (Se dopant)	GaInP n-type
p GaInP (Zn dopant)	GaInP p-type
p+ AlGaInP	window (front passivation) for GaInP cell
p++ AlGaAs /GaAs	contact layer (**T contact**); this layer is etched away between the T-contact grids before being epoxied to the glass superstrate

The 3TT devices were fabricated according to the process in **Figure 2b**. Photolithography was used throughout the fabrication process to define etch areas. The processing started with

electroplating a gold front contact grid to the p++ AlGaAs/GaAs layer of the epitaxially grown sample (the T contact). The uncontacted portion of the p++ AlGaAs/GaAs layer was then removed with 2:1:10 (by volume) $NH_4OH:H_2O_2:H_2O$, in order to prevent parasitic optical losses (step 2). The sample was bonded with transparent epoxy to a glass superstrate handle (step 3). Next, the GaAs substrate on which the device was grown was etched away with 1:3 (by volume) $NH_4OH:H_2O_2$ solution and then the GaInP stop etch layer was removed with HCl, exposing the n++ GaInAsN layer which is the location of the R contact (step 4).

At step 5 the procedure was changed based on the back contact gold deposition method used for the sample (electrodeposition or evaporation); **Figure S2** shows the differences in processing between these two methods. For electrodeposited gold, the R contact was patterned and gold was electrodeposited onto it (5a). The GaAs subcell mesa was then patterned with photoresist and isolated via etching (using HCl for phosphide layers and 3:4:1 (by volume) $H_3PO_4:H_2O_2:H_2O$ for arsenide layers) to reveal the p-type side of the tunnel junction (p++ AlGaAs) (5b). The tunnel junction is sufficiently narrow to allow e^- extraction even though the p-type side is contacted. Gold was then electroplated onto the p++ AlGaAs to form the Z contact (5c) and the same selective etchants as the first mesa were used to finish mesa isolation around the border of the Z and R contacts, revealing the T contact (5d). For evaporated gold, the GaAs subcell was isolated as above to reveal the Z contact layer (5a). The full mesa was etched to reveal the T contact around the border of the device (5b). Then both the Z and R contacts were patterned for evaporation and 10 nm of nickel and then 300 nm of gold were deposited using a Temescal FC2000 E-beam evaporator depositing nickel at 3 Å/s and gold at 10 Å/s (5c). All remaining steps (steps 6-9) were the same for all devices. For step 6, SU-8 (a dielectric photoresist epoxy) was applied along the mesa borders via inkjet printing followed by a 5-minute UV cure then a 5-minute bake at 110°C. The *I-V* characteristics of all devices were measured before further processing.

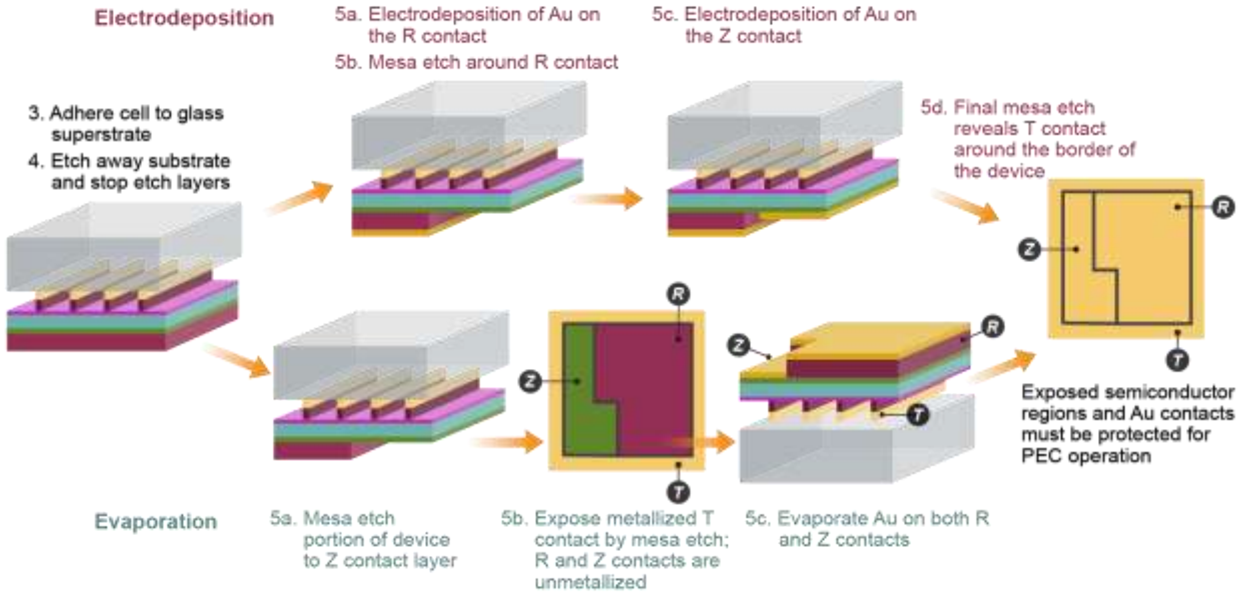

Figure S2: 3TT fabrication procedure with gold deposition details for electrodeposition or evaporation methods. Illustration by Al Hicks, NREL.

Dry Device Characterization

3TT and 2TT devices were characterized with quantum efficiency (QE) and current-voltage (I-V) measurements. **Table S2** shows the characteristics of each 3TT and 2TT device used in PEC experiments. As described in the main text, sample numbers are assigned based on MOVPE deposition run at the National Renewable Energy Laboratory (NREL). Growth run numbers (MUXXX or MVXXX) are used in the main text; nX designators used here identify specific devices from a growth, distinguished in the NREL database. Only one sample with electroplated gold (MU845) was used in this study due to the reduced amount of shunting in samples with evaporated gold. Dry *I-V* measurements were either calibrated to 1 Sun (AM 1.5 G) or slightly over-illuminated (MU845), using calibrated GaInP and GaAs reference cells to set the intensity. Because the GaInP subcell is under-illuminated when the GaAs subcell is at 1 Sun, a blue LED is used in addition to the xenon lamp for calibrated *I-V* measurements.[4] LEDs were not used during light calibration for PEC. Although MU845 was slightly over-illuminated and did not have a bias LED, there should be minimal impact on measured open-circuit potential, V_{OC}, and short-circuit current, I_{SC}, will be a slight underestimation. A two-terminal measurement of the 2TT device is equivalent to a TR measurement on a 3TT.

Table S2: Summary of fabrication and dry characterization values for each sample tested; full sample names are given (short names used in the main text). *Data from MU813 is only reported in **Figure S3** to illustrate the connection between the *I-V* measurements and power contour plots used to characterize 3TT devices outside of this work; it was not fabricated into a full CoPc/MWCNT photocathode.

		**3TT Devices**						**2TT Device**
**Sample**		MU845n2p	MV176n1	MV117n2	MV074n1	MV063n1	MU813n4*	MV337n2
**Gold Deposition Method**		Electroplated	Evaporated	Evaporated	Evaporated	Evaporated	Electroplated	Evaporated
**I-V Illumination**		Over illuminated, no bias LED	1 Sun	1 Sun	1 Sun	1 Sun	1 Sun	1 Sun
**TR**	$V_{\mathrm{OC}}^{\mathrm{TR}}$ **(V)**	2.09	2.30	2.26	2.28	2.28	2.33	2.22
	$I_{\mathrm{SC}}^{\mathrm{TR}}$ **(mA)**	8.05	6.70	6.74	7.09	6.86	7.77	10.19
	FF_{TR} **(%)**	53.61	87.79	77.05	66.61	86.97	90.30	60.87
**TZ**	$V_{\mathrm{OC}}^{\mathrm{TZ}}$ **(V)**	1.10	1.34	1.32	1.32	1.31	1.33	--
	$I_{\mathrm{OC}}^{\mathrm{TZ}}$ **(mA)**	11.03	12.61	12.12	11.74	11.76	10.80	--
	FF_{TZ} **(%)**	55.39	81.00	80.32	74.97	73.30	86.53	--
**ZR**	$V_{\mathrm{OC}}^{\mathrm{ZR}}$**(V)**	0.96	0.96	0.94	0.94	0.94	0.99	--
	$I_{\mathrm{SC}}^{\mathrm{ZR}}$ **(mA)**	7.48	6.74	6.75	6.93	6.88	7.89	--
	FF_{ZR} **(%)**	38.15	79.07	66.65	50.84	74.32	84.78	--

A circuit model of the 3TT device structure is shown in **Figure S3a**. Generally, 3TT devices should be characterized using power contour plots rather than simple current-voltage analysis to fully capture device behavior across all degrees of freedom.[1,5] **Figure S3b** shows *I-V* curves and **Figure S3c** shows a power contour plot measurement for one wiring configuration of a 3TT device measured on voltage-voltage axes (out of six possible equivalent presentations).[6] However, 3TT photocathodes will have reduced degrees of freedom during operation when both

Z and R contact the electrolyte. Quantum efficiency was measured on a custom-built instrument with a tungsten-halogen lamp and a 270m monochromator. The devices used to measure QE were non-inverted 2TT test structures fabricated from the same growth structure (NREL sample MV006) and processed with electroplated Au/Sn/Au back contacts on the substrate side and electroplated Au grids on the T contact side. High brightness LEDs at 470 nm and 850 nm were used to separate the GaInP and GaAs cell responses. The quantum efficiency results were used in the light calibration processes for *I-V* measurements.[7] The 3TT design does not incorporate an explicit dielectric anti-reflective coating, though the superstrate glass in the 3TT does reduce some reflection losses. The 2TT test structure used for quantum efficiency measurements does not include the glass and so the measured quantum efficiency is slightly lower than in the 3TT, although the shape is the same.

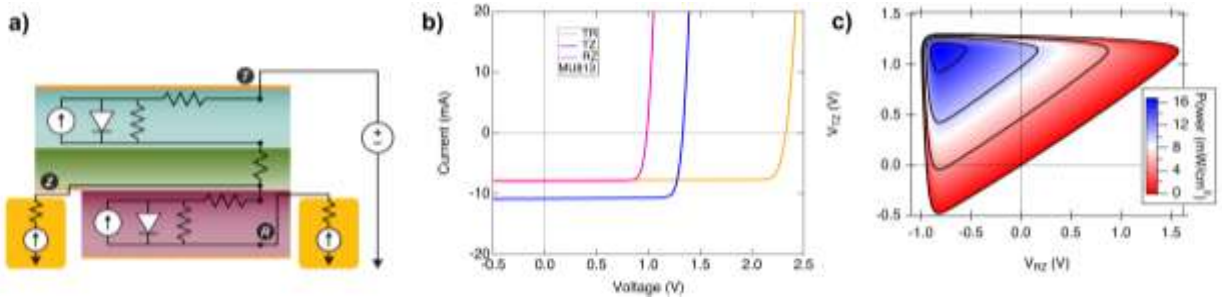

Figure S3: a) circuit diagram of a 3TT photocathode device; b) *I-V* curves and c) power contour plot of a representative 3TT device (NREL sample MU813) showing one possible wiring configuration.[6]

<u>Continuum Model for (Photo)electrochemical Operating Conditions</u>

Steady-state mass conservation and Fick's law of diffusion were used to solve for the concentration profiles of CO_2 and CO within the electrolyte boundary layer adjacent to the cathode surface (model is illustrated in **Figure S4**).

$$D_i \nabla^2 c_i + u \cdot \nabla c_i = 0 \qquad (\mathbf{S1})$$

where D_i and c_i are the diffusivity and concentration of species i, respectively, and u is the electrolyte velocity. The first term describes diffusion, and the second term describes convection. The diffusivities and other model parameters are provided in **Table S3**.

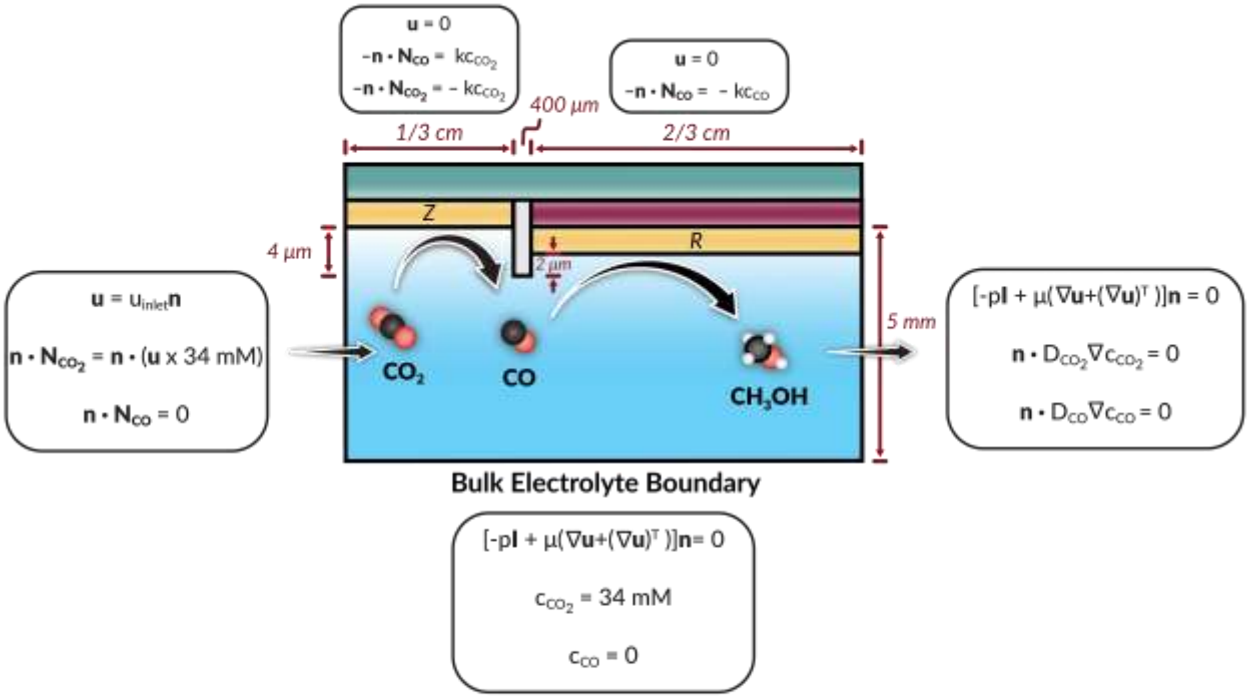

Figure S4: Illustration of model domain and the mathematical form of all the boundary conditions.

Table S3: List of continuum flow model parameters.

Parameter	Value	Units	Ref.
D_{CO2}	1.92×10^{-5}	cm^2 s^{-1}	[8]
D_{CO}	2.03×10^{-5}	cm^2 s^{-1}	[8]
$L_{electrolyte}$	5	mm	-
k	10^{-5}	$m\ s^{-1}$	fit

The velocity profile was calculated by the continuity equation and the Navier-Stokes equation for an incompressible, Newtonian fluid:

$$\rho \nabla \cdot u = 0 \tag{S2}$$

$$\rho(u \cdot \nabla)u = \nabla \cdot [pI + \mu(\nabla u + (\nabla u)^T)] \tag{S3}$$

where ρ and μ are the density and dynamic viscosity of water, respectively (used material property value for water within COMSOL at 20°C), p is pressure, and I is the identity matrix. **Equations S1-S3** were solved simultaneously within COMSOL Multiphysics 6.0 software with a non-uniform triangular mesh that was fine at the two terminals and coarse in the bulk of the electrolyte. A sensitivity analysis on the mesh identified a minimum size of 10085 node points. The boundary conditions are presented in **Figure S4**. In summary, a no slip condition was used at the catalyst surfaces, the bulk electrolyte boundary was treated as an open boundary, and inflow and outflow conditions were used at the entrance and exit, respectively. The surface reactions were modeled with simple first-order rate expressions where the rate constant (k) was fit so that the simulated methanol current density versus inlet flow rate peaks around the experimentally

measured optimal inlet flow rate (~ 8.5 cm/min) for a single terminal device.[9] In general, changing k changes the inlet flow rate that maximizes methanol production.

The predicted CO concentration profile at an inlet flow rate of 8.5 cm/min is presented in **Figure S5a**, showing that the concentration of CO is relatively high across the R terminal. We note that the local CO concentration is predicted to significantly exceed the solubility limit of CO in water (~1 mM),[10] which is consistent with previous modelled and experimental work on an electrochemical cascade with a CO intermediate where local supersaturation of CO was observed.[11] This high local CO concentration should promote the reduction of CO to methanol by the law of mass action. Changing the inlet flow rate alters the local CO concentration across the R terminal and, thereby, the methanol generation rate, as seen in **Figure S5b**. The optimal inlet flow rate (8.5 cm/min) is the same as previously reported for a flat catalytic surface,[9] but the peak methanol current density for this 3TT geometry is much narrower in terms of flow rate, because the 2 µm SU-8 step between the terminals is an impenetrable wall that forces CO away from the surface, leading to significant amounts of CO being swept out the system by convection at inlet flow rates higher than the optimal. Inlet flow rates below the optimal lead to low average CO concentrations across the R terminal, and therefore low currents for production of methanol.

Surface plots of modeled CO_2 and CO concentrations at different electrolyte flow rates are shown in **Figure S6a-e.** The inlet flow rate affects the local concentration of dissolved CO_2 as well as the concentration of CO (**Figures S6a-c**), but CO_2 concentration has a negligible effect on the rate of the overall cascade reaction because the conversion of CO to methanol is the rate limiting step. Below ~ 8.5 cm/min, the generated CO remains close to the R terminal (**Figures S6d** and **e**), but the local concentration of CO is maximized around 8.5 cm/min. Beyond this optimal flow rate, the rate of convection is too rapid and CO is swept out of the system before it can be converted to methanol (**Figure S6f**). Thus, inlet electrolyte flow rate is a critical parameter for controlling the transport of unbound CO and, thereby, the production of methanol. We note that this model does not correctly predict methanol generation rate at an inlet velocity of zero because the model is of a flow cell system and, hence, we restrict the simulated flow rates to above zero. Moreover, the experimentally measured methanol current density in this work is higher than that predicted by the model because the model assumes the catalyst is a flat surface, when experimentally the CoPc/MWCNT catalyst is a porous layer. Nonetheless, the model helps to reveal how the CO intermediate transports between terminals and how the inlet flow rate modulates this process.

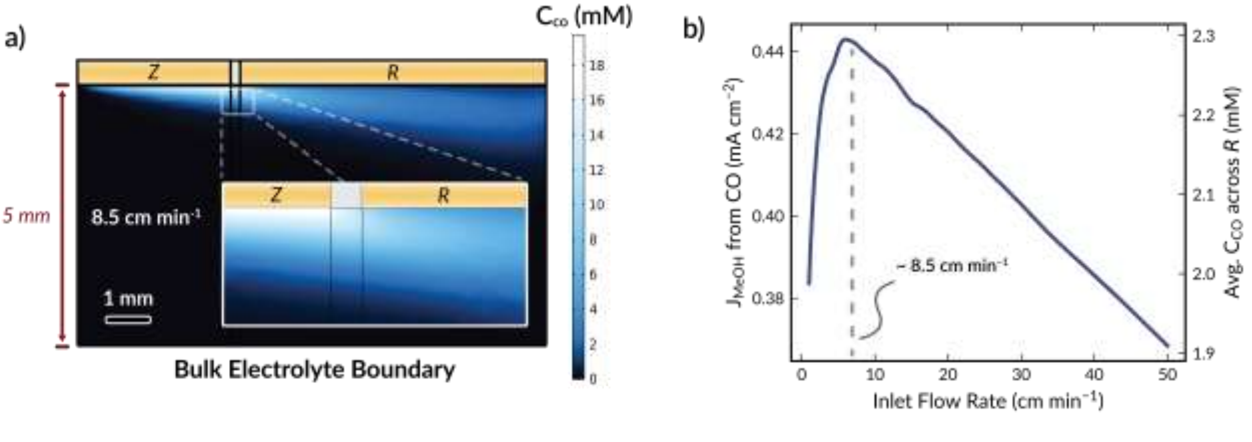

Figure S5: a) Surface plot of CO concentration (C_{CO}) throughout the full model domain at an inlet flow rate of 8.5 cm/min; figure inset is the C_{CO} near the junction of the two terminals. b) Simulated methanol current density (j_{MeOH}) from CO reduction and the average C_{CO} across the R terminal as a function of inlet flow rate.

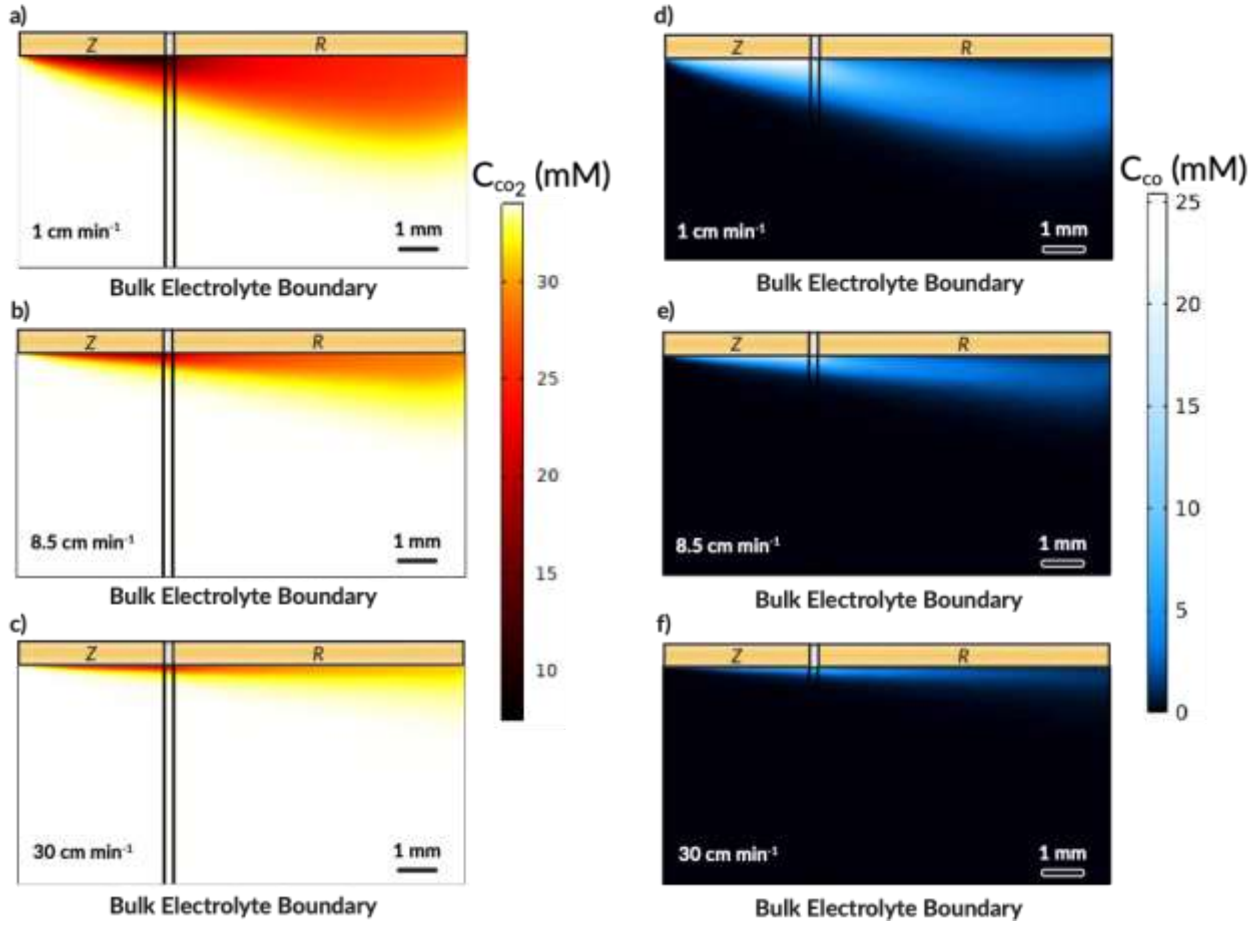

Figure S6: Surface plots of reactant concentrations throughout the model domain at varied electrolyte inlet velocities. CO_2 concentrations at a) 1 cm/min, b) 8.5 cm/min, c) 30 cm/min; the maximum concentration of CO_2 is 34 mM. CO concentrations at d) 1 cm/min, e) 8.5 cm/min, f) 30 cm/min.

CO_2R CoPc/MWCNT Catalyst

Catalyst Materials

The multiwalled carbon nanotubes (MWCNTs, TEM 12 nm x 10 µm, 98% Sigma-Aldrich) were washed in HPLC grade HCl (Sigma-Aldrich 99.999% purity). The catalyst used was cobalt phthalocyanine (CoPc, Thermo Fischer, purity > 95%). Dispersions of CoPc and MWCNTs were made in isopropyl alcohol (Sigma-Aldrich ≥ 99.5%). For an adhesion layer, 3-4% (wt, in H_2O) high conductivity poly(3,4-ethylenedioxythiophene) polystyrene sulfonate (PEDOT:PSS, > 200 S/cm, Sigma-Aldrich) was used. The ink was prepared by following the procedure from a previous report;[9] the catalyst ink suspension was created by combining MWCNTs and CoPc in a 1:0.6 mass ratio and probe sonicating (Cole-Parmer 500 W probesonicator) in isopropanol in a 1 mg/ml ratio.

Catalyst Layer Deposition

For the dark experiments, 2.5 cm x 2.5 cm glass slides were cleaned via sonication for 10 minutes in acetone, soap water, DI water, and isopropyl alcohol in that order and then dried under N_2 flow. While 3TTs have a separation between Z and R contacts of 200 - 400 μm, for these dark experiments a very thin strip of copper tape (1 mm) was placed down the middle and was used as a mask, leading to a slightly larger separation between Z_{dark} and R_{dark}. Then 10 nm of Ta was sputtered onto the glass slides (AJA Magnetron Sputtering) at 150 W and 3 mTorr Ar (deposition rate: 0.8 Å/s) followed by 150 nm of Au under the same conditions (2.9 Å/s). The targets were located off center with respect to the sample stage, which was rotated at 100 rpm. A quartz crystal monitor was used to measure the deposition rates. PEDOT:PSS layers and catalyst layers were deposited in the same way and same loading to the 3TT photocathode, resulting in devices such as the one pictured in **Figure S7**.

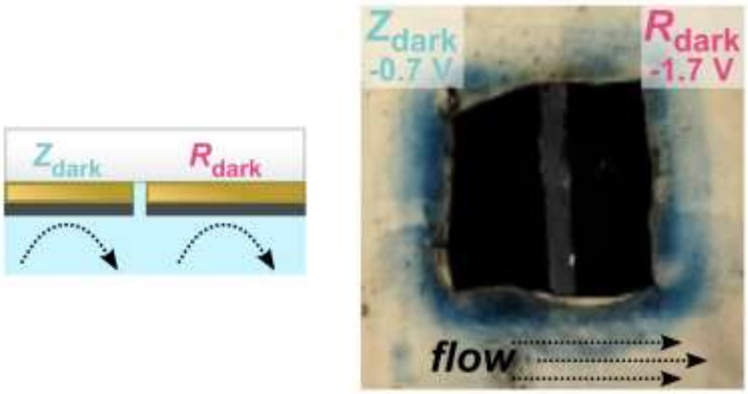

Figure S7: Schematic (left) and photograph (right) of the two-part electrode used to determine operating potentials for the 3TT photocathode. For the tested devices, Z_{dark} had an average area of 0.55 cm^2 and R_{dark} had an average area of 0.36 cm^2.

For 3TT devices, custom shadow masks were made to cover one contact at a time in order to selectively deposit PEDOT:PSS and catalyst on each contact (Z and R) without shorting. Shadow masks were aligned by hand and affixed with tape, a stereoscopic microscope was used to check alignment. With one contact exposed, a solution of PEDOT:PSS (a 10 fold dilution of 3.0-4.0% (wt, in H_2O) high conductivity PEDOT:PSS) was sprayed onto the solar cell using an ultrasonic spray coater (Sonotek ExactaCoat) or sprayed by hand with a Master Airbrush Model G79 airbrush, referred to as “ultrasonic” or “airbrush” deposition methods respectively. The ultrasonic method was performed at a flow rate of 0.05 mL/min and a run power of 1.8 W with the stage held at 95°C, spraying over a 2.5 x 2.5 cm square and sprayed 630 μL, leading to a PEDOT:PSS loading of 10 μL cm^{-2} of the 3-4% solution. The airbrush method was performed with the sample on a 95°C hot plate. Cross-sectional SEM was performed on a representative blank sample (a glass substrate with 10 nm of Ti, 100 nm of Au that was spray coated with PEDOT:PSS) to determine that the PEDOT:PSS layer was approximately 2 μm. The same procedure was repeated with the other mask on the other contact. A similar procedure was used for coating the catalyst except the ultrasonic stage or hot plate was held at 85°C and the ultrasonic method used a

flow rate of 0.1 mL/min and a run power of 2.1 W. We sprayed ~2.5 mg of catalyst material over the 6.25 cm^2, leading to a CoPc/MWCNT loading of ~0.4 mg cm^{-2} and a CoPc loading of ~0.15 mg cm^{-2}. **Figure S8** shows a 3TT photocathode coated with PEDOT:PSS and CoPc/MWCNT.

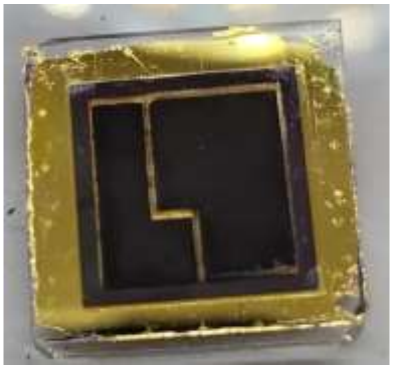

Figure S8: Photograph of 3TT with all catalyst layer coatings (viewed from electrolyte contact side). The R contact is on the right and larger than the Z contact, and the T contact is visible around the border of the device. The final footprint of the III-V device (within the black border in the photograph) is 1.2 cm x 1.2 cm; the glass is 1.7 cm x 1.7 cm.

Cascade (Photo)electrochemistry

Experimental Setup

The three-electrode flow cell used to test both dark electrodes and 3TT photocathodes was identical to that in our previous report, including electrolyte flow and gas connections.[9] Dark electrochemical measurements were performed using a two channel SP-300 Biologic bipotentiostat, with the working electrode of one channel connected to R_{dark} and the other connected to Z_{dark}. The counter senses for the two channels were connected and the reference cables for the two channels were also connected, enabling the use of one counter and one reference electrode in the cell with two working electrodes. Synchronized chronoamperometry (CA) could then be performed for dark tandem experiments.

A Selemion membrane (AGC Engineering) was used to separate the anode and cathode chambers. Because of the focus on the reduction half cell in this study, Carbon Fiber Paper Torray 120 (Fuel Cell Store) was used as the anode (counter electrode, 1 cm x 1 cm). In order to avoid contamination of the working electrode from metal dissolution and crossover,[12,13] no oxygen evolution reaction catalyst was used at the counter electrode. While this likely results in an overall cell potential drop which can be avoided in a fully optimized cell by employing a catalyst, these measurements are performed in a three-electrode cell where anode potential drop is not a major issue. An eDAQ leakless Ag/AgCl aqueous reference electrode was used as the reference electrode (5 mm from working electrode) and stored in a saturated KCl solution. Copper tape was applied to the 3TT T contact (or the base gold layer for the electrochemical devices) to allow for connection to the potentiostat as the working electrode, then any exposed gold was protected with insulating electrochemical tape (3M). Potassium carbonate (99% purity, Sigma-Aldrich) and potassium bicarbonate (99.7% purity, Sigma-Aldrich) were saturated with CO_2 and used as the electrolyte.

Illumination

3TT photocathodes were illuminated from the T contact side (opposite to the contact with the electrolyte). The photoelectrochemical cell was illuminated with a Xe arc lamp (Solar Light 300W Air-Mass 1.5 Solar Simulator, 16S-300-2.2-AM) with a water filter attached. A pyranometer was used to align the 3TT photocathode to ensure it was exposed to a fixed light intensity of 1 Sun (100 mW cm^{-2}) or 1.1 Sun. LEDs were not used during PEC light calibration, so 1 Sun illumination to both subcells simultaneously was not possible.[4] Additionally, pyranometers measure power and not wavelengths of light, so the illumination spectrum was not explicitly measured during calibration. The only difference from the previously published electrochemical cell setup[9] was that some measurements had a 1 x 1 cm^2 hole cut into the center of the polycarbonate backplate that holds the working device to the flow cell to prevent parasitic light absorption from the polycarbonate backing the 3TT photocathode (**Figure S9**).

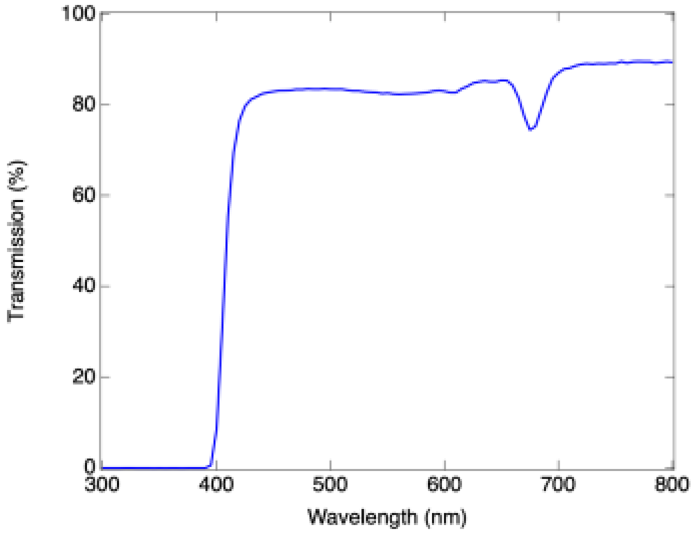

Figure S9: UV-Vis transmission of the polycarbonate mounting plate; the polycarbonate is highly absorbing below 400 nm, blocking some light to the GaInP subcell.

Determination of T Contact Operating Potentials for Chronoamperometry

Photoelectrochemical CA measurements were performed with a single channel of the SP-300 Biologic potentiostat, where the working electrode was the T contact of the 3TT photocathode. As described in the main text, the target voltage for CA (V_{CA}) is where the R contact is at –1.7 V vs RHE, based on the dark control experiments. In our previous work with CoPc/MWCNT catalysts[9] the target voltage was –1.2 V vs RHE, with solution resistance corrected using current-resistance (iR) compensation by a 50 Ω resistance. Without iR compensation, the target was –1.7 V vs RHE, consistent with the dark electrochemical result presented in this work. For some replicate CoPc/MWCNT 3TT photocathode experiments, V_{CA} was slightly varied from the calculated V_{CA} value to explore different potentials given the inherent uncertainty in the approximation used to determine this value, as discussed in the main text. V_{CA} values are listed in **Table S4**. The electrode used to obtain dark $E_{1/2}$ was a simple glass slide with gold, PEDOT:PSS

and CoPc/MWCNT made according our prior work.[9] To obtain $E_{1/2}$ values, the reduction of CoPc (Co^{II}/Co^{I}) in 0.1 M $KHCO_3$ or methyl viologen (MV^{2+}/MV^{+}, 5 mM methyl viologen) was performed.[14] $E_{1/2}$ values were then compared to those determined by operation of the CoPc/MWCNT 3TT photocathode.

Product Detection

For the gaseous products, the same product quantification method was used from our prior work, where we obtained the gas product concentrations over time using a SRI Multi Gas 3 gas chromatograph (GC).[9] The outlet of the catholyte chamber was connected to the inlet of the GC to allow for gas detection. The GC was equipped with a 2 mL sampling loop that injects the gas every 16.5 minutes. Two columns (Hayesep-D) and Mol Sieve 13x were used to separate the gases, and an electronic valve was used to redirect the bulk of the CO_2 band as to not overwhelm the detectors. A methanizer and flame ionizing detector was used for CO detection, and a thermal conductivity detector was used for H_2 detection. Argon was used as the carrier gas.

The following formula was used to calculate gas product Faradaic efficiency:

$$FE(\%) = \frac{C_{prod} \cdot J \cdot n \cdot F}{i} \times 100 \qquad \textbf{(S4)}$$

where C_{prod} is the concentration of the product by GC, J is the molar flow rate of CO_2 into the reservoir, n is the number of electrons to the product (2 for both H_2 and CO), F is Faraday's constant, and i is the average current at time of injection, time averaged in a 3-minute window, which corresponds approximately to the total mixing volume of the system. To calculate the error, six gas measurements were taken over the course of the 80-minute experiment. The first gas measurement is excluded from analysis as it is taken at the start (t= 0 min). The error of the gas measurements was calculated from the standard deviation of all the gas measurements from the experimental condition.

Liquid products were quantified using a Bruker 500 MHz nuclear magnetic resonance (NMR) system. The same procedure for NMR quantification was used from our prior work,[9] based on the procedure from Chatterjee et al.[15] An internal standard of 50 mM and 50 mM phenol in DI water was prepared. Each NMR sample tube was prepared with 400 µL analyte, 50 µL internal standard, and 50 µL D_2O. The 90° pulse width was obtained for each solvent system and the center of the water peak. A solvent suppression method was then used to obtain the spectrum using a 25 second delay time. The integration ratio between the methanol peak and a dimethyl sulfoxide (DMSO) peak was used to calculate the methanol concentration. Calibration of the internal standard was performed using different standard solutions of methanol. Once the concentrations of species were calculated, the concentrations were used to determine the Faradaic efficiencies.

The following formula was used to calculate the liquid product FE:

$$FE(\%) = C_{ref} \cdot \frac{I_P}{I_R} \cdot \frac{H_R}{H_P} \cdot \frac{nFV}{Q} \cdot \frac{5}{4} \times 100 \qquad \textbf{(S5)}$$

where C_{ref} is the concentration of the reference, I_P and I_R are the integrations of the product and reference respectively, H_R and H_P are the number of corresponding protons on the reference and product respectively (6 for DMSO, 3 for methanol), n is the number of electrons to product (6 for methanol), F is Faraday's constant, V is the volume of electrolyte used for the experiment (10 mL

typically), Q is total charge passed, and the factor of 5/4 is due to 400 µL of analyte being diluted to 500 µL for the NMR analysis. The error of the liquid product FEs were calculated from the standard deviation of three individual measurements of the same liquid sample.

Table S4 summarizes all measurements of 3TT photocathodes for this study, including Faradaic efficiency (FE) towards products made, V_{CA} values, catalyst deposition method, and other variations between samples and experiments. All CAs are 80 minutes long for 3TT photocathodes and 40 minutes long for the 2TT photocathode (MV337).

Table S4: Summary of photoelectrochemical characteristics and setup specific to each 3TT sample, including the products made, V_{CA} values, illumination conditions, and catalyst deposition method.

PV Sample	Illumination Conditions	Illuminated Through Polycarbonate	$V_{OC}^{TR,PEC}$ (V)	V_{CA} (V vs RHE)	V_{CA} Method	FE Methanol (%)	FE CO (%)	FE H_2 (%)	I_{avg} (mA)	Catalyst	Catalyst Dep. Method
**MU845**	1.1 Sun	Yes	2.0	+0.30	CoPc	3.8 ± 0.4	65 ± 9	32 ± 1	4.1 ± 0.2	Pristine	Airbrush
	1.1 Sun	Yes		–0.20		0.9 ± 0.2	48 ± 5	54 ± 11	6.3 ± 0.1	2nd Run	
	1.1 Sun	Yes		0.00		2.9 ± 0.3	38 ± 1	69 ± 2	5.5 ± 0.2	3rd Run	
	1.1 Sun	Yes		+0.30		0.9 ± 0.5	90 ± 6	19 ± 1	3.7 ± 0.1	Regen	Dropcast
	1.1 Sun	Yes		+0.20		1.5 ± 0.4	79 ± 2	27 ± 2	4.6 ± 0.1	2nd Run	
	1.1 Sun	Yes		+0.20		1.6 ± 0.3	61 ± 5	28 ± 1	5.4 ± 0.3	Regen	Dropcast
	1.1 Sun	Yes		+0.20		1.5 ± 0.3	38 ± 3	66 ± 3	5.8 ± 0.5	Regen	Dropcast
	1.1 Sun w/ filter	Yes		+0.20		0.0	70 ± 9	40 ± 2	1.51 ± 0.06	2nd Run	
**MV063**	1.1 Sun	Yes	2.3	+0.60	CoPc	0.7 ± 0.3	14 ± 5	86 ± 5	6.3 ± 0.2	Pristine	Ultrasonic
**MV074**	1.1 Sun	Yes	2.25	+0.65	CoPc	2.1 ± 0.5	37 ± 10	63 ± 15	3.5 ± 0.2	Pristine	Ultrasonic
	1.1 Sun w/ filter	Yes		+0.65		0.0	90 ± 6	26 ± 3	0.75 ± 0.05	2nd Run	
**MV176**	1 Sun	No	1.65	–0.25	MV	0.8 ± 0.6	17 ± 3	85 ± 7	6.9 ± 0.5	Pristine	Ultrasonic
**MV117**	1 Sun	No	2.25	+0.40	MV	1.4 ± 0.8	79 ± 4	38 ± 4	4.7 ± 0.3	Pristine	Ultrasonic
**MV337 (2TT)**	1 Sun	Yes	1.4	–0.3	CoPc	5 ± 2	44 ± 4	53 ± 7	3.7 ± 0.4	Pristine	Airbrush

Regeneration of CoPc Catalyst

The catalyst film on the 3TT photocathodes was carefully regenerated by the following procedure. The 3TT sample was rinsed carefully with DI water to remove residual electrolyte salt, and then left to dry on a glass petri dish heated to 85°C on a hot plate. The existing catalyst film was not removed, but regenerated by using the same ink formulation, and dropcasting 10 µL at a time onto the 3TT device on an 85°C hot plate, taking care not to cause any shorts between the R, Z, or T contacts ("dropcast" in **Table S4**). A total volume of 500 µL of catalyst ink was drop casted onto the sample and left to dry for 1 hour to remove all residual solvent from the sample. Any catalyst ink dropped on the middle line was wiped away carefully with a small cotton swab. The PEDOT:PSS adhesion layer was not regenerated in this regeneration procedure. Figure S12 shows the CAs of regenerated 3TT photocathodes. **Figure S10** shows the CAs for MU845 after each regeneration of catalyst.

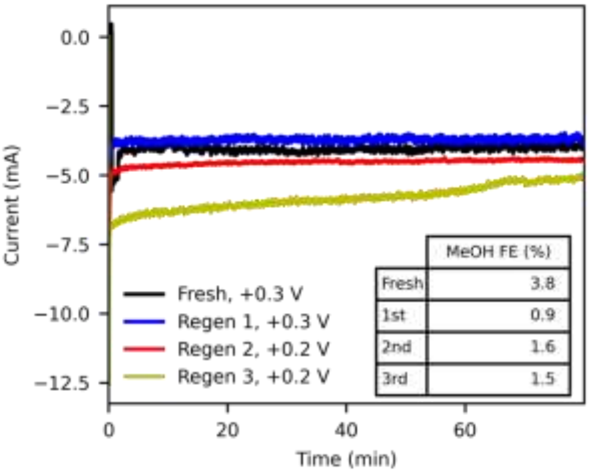

Figure S10: Chronoamperometry experiments of each regeneration of the CoPc/MWCNT 3TT photocathode MU845.

Light Filter Experiments

AM 1.5 illumination was filtered using a 650 nm short-pass filter from Thorlabs. The transmission spectrum of the filter is shown in **Figure S11a**, and representative *I-V* curves (dry) of a 3TT device with and without the filter are shown in **Figure S11b**. The short-pass filter effectively zeroes the current in the ZR and TR measurements, indicating that no light reaches the GaAs subcell.

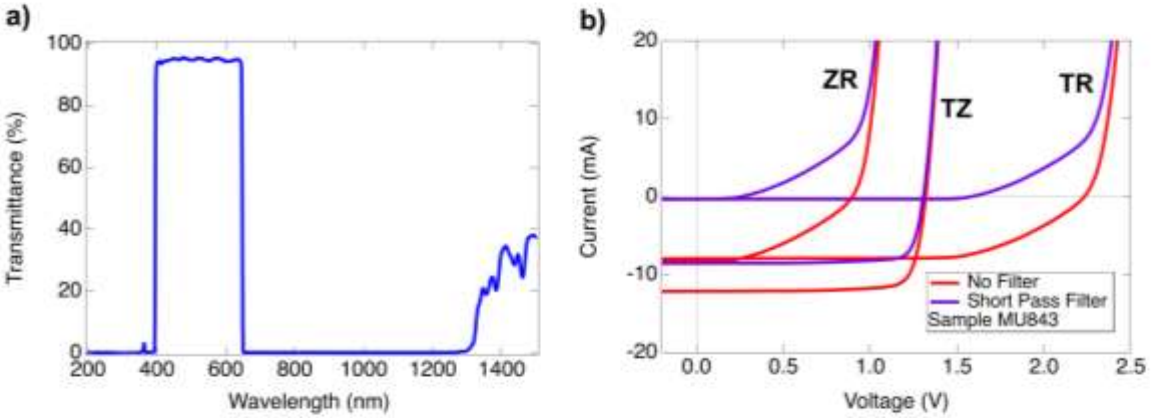

Figure S11: a) Transmission spectra of the short-pass filter (compare to the quantum efficiency of the subcells in Figure 2c). b) Dry *I-V* curves of a 3TT sample under AM 1.5G illumination (red) and with the filter in place (purple). With the short-pass filter in place, the GaAs subcell (ZR) passes no current, while GaInP (TZ) is only slightly reduced in current. The tandem (TR) also passes no current, as current through the two series-connected subcells is limited by the lower current subcell.

References

(1) Warren, E. L.; McMahon, W. E.; Rienäcker, M.; VanSant, K. T.; Whitehead, R. C.; Peibst, R.; Tamboli, A. C. A Taxonomy for Three-Terminal Tandem Solar Cells. *ACS Energy Lett.* **2020**, *5*, 1233–1242.

(2) Nielander, A. C.; Shaner, M. R.; Papadantonakis, K. M.; Francis, S. A.; Lewis, N. S. A Taxonomy for Solar Fuels Generators. *Energy Environ. Sci.* **2015**, *8*, 16–25.

(3) Steiner, M. A.; Geisz, J. F.; García, I.; Friedman, D. J.; Duda, A.; Kurtz, S. R. Optical Enhancement of the Open-Circuit Voltage in High Quality GaAs Solar Cells. *J. Appl. Phys.* **2013**, *113*, 123109.

(4) R. Osterwald, C. IV-1 - Standards, Calibration and Testing of PV Modules and Solar Cells. In *Practical Handbook of Photovoltaics*; Markvart, T., Castañer, L., Eds.; Elsevier Science: Amsterdam, 2003; pp 793–816.

(5) Kong, C. J.; Warren, E. L.; Greenaway, A. L.; Prabhakar, R. R.; Tamboli, A. C.; Ager, J. W. Design Principles of Tandem Cascade Photoelectrochemical Devices. *Sustain. Energy Fuels* **2021**, *5*, 6361–6371.

(6) Geisz, J. F.; McMahon, W. E.; Buencuerpo, J.; Young, M. S.; Rienäcker, M.; Tamboli, A. C.; Warren, E. L. Characterization of Multiterminal Tandem Photovoltaic Devices and Their Subcell Coupling. *Cell Rep. Phys. Sci.* **2021**, *2*, 100677.

(7) Emery, K.; Meusel, M.; Beckert, R.; Dimroth, F.; Bett, A.; Warta, W. Procedures for Evaluating Multijunction Concentrators. In *Conference Record of the Twenty-Eighth IEEE Photovoltaic Specialists Conference - 2000 (Cat. No.00CH37036)*; 2000; pp 1126–1130.

(8) Cussler, E. L. *Diffusion: Mass Transfer in Fluid Systems*, 3rd ed.; Cambridge University Press: Cambridge, 2009.

(9) Chan, T.; Kong, C. J.; King, A. J.; Babbe, F.; Prabhakar, R. R.; Kubiak, C. P.; Ager, J. W. Role of Mass Transport in Electrochemical CO_2 Reduction to Methanol Using Immobilized Cobalt Phthalocyanine. *ACS Appl. Energy Mater.* **2024**, *7*, 3091–3098.

(10) Gevantman, L. H. *CRC Handbook of Chemistry and Physics*, 90th ed.; CRC Press: Boca Raton, FL.

(11) Gurudayal; Perone, D.; Malani, S.; Lum, Y.; Haussener, S.; Ager, J. W. Sequential Cascade Electrocatalytic Conversion of Carbon Dioxide to C–C Coupled Products. *ACS Appl. Energy Mater.* **2019**, *2*, 4551–4559.

(12) Cherevko, S.; Geiger, S.; Kasian, O.; Kulyk, N.; Grote, J.-P.; Savan, A.; Shrestha, B. R.; Merzlikin, S.; Breitbach, B.; Ludwig, A.; et al. Oxygen and Hydrogen Evolution Reactions on Ru, RuO_2, Ir, and IrO_2 Thin Film Electrodes in Acidic and Alkaline Electrolytes: A Comparative Study on Activity and Stability. *Catal. Today* **2016**, *262*, 170–180.

(13) Jerkiewicz, G. Applicability of Platinum as a Counter-Electrode Material in Electrocatalysis Research. *ACS Catal.* **2022**, *12*, 2661–2670.

(14) Elgrishi, N.; Rountree, K. J.; McCarthy, B. D.; Rountree, E. S.; Eisenhart, T. T.; Dempsey, J. L. A Practical Beginner's Guide to Cyclic Voltammetry. *J. Chem. Educ.* **2018**, *95*, 197–206.

(15) Chatterjee, T.; Boutin, E.; Robert, M. Manifesto for the Routine Use of NMR for the Liquid Product Analysis of Aqueous CO2 Reduction: From Comprehensive Chemical Shift Data to Formaldehyde Quantification in Water. *Dalton Trans.* **2020**, *49*, 4257–4265.
